# Buried soils from the Holocene Humid Period in Wadi Shuwayhi, Al-Khashbah (Oman)

**DOI:** 10.1038/s41598-026-55740-1

**Published:** 2026-07-07

**Authors:** Dana Pietsch, Tara Beuzen-Waller, Conrad Schmidt, Katharina E. Schmitt, Peter Kühn

**Affiliations:** 1 Research Area Geography, Chair of Soil Science and Geomorphology, University of Tübingen, Tübingen, Germany; 2 Institute of Geography, University of Heidelberg, Heidelberg, Germany; 3 UMR 7194 HNHP—PAST, University of Perpignan Via Domitia, Perpignan, France; 4 Institute for Ancient Near Eastern Studies (IANES), University of Tübingen, Tübingen, Germany; 5 Institute of Geosciences, Applied and Analytical Paleontology Work Group, Johannes Gutenberg University Mainz, Mainz, Germany

**Keywords:** Paleosols, Stratigraphy, Holocene Humid Period, Central Oman, Climate sciences, Ecology, Ecology, Environmental sciences, Solid Earth sciences

## Abstract

The paleosol horizons on the lower terrace of Wadi Shuwayhi are the first buried soils from the Holocene Humid Period in Central Oman have been studied; they occur in alluvial sediments (terrace fill) deposited approximately 11.5 to 7 ka, with radiocarbon ages of soil organic matter range from 10,600 to 5300 cal BP (8700–3500 cal BC). The youngest buried soil horizon in profile KS3 show disturbances in soil structure, consistent with Early Bronze Age cultivation at 4800–4400 cal BP (2800–2500 cal BC). Pedogenic features in the buried A and B horizons of all paleosols show subangular blocky or crumb macro- and microstructure, bioturbation features, and secondary calcification within root channels. In two soils, tabular and lenticular gypsum is particularly pronounced. Although the organic carbon content is low at all sites, soil formation suggests earlier vegetation establishment during the Early Holocene as is also known from other arid areas. Alternating phases of alluvial aggradation and lateral erosion, i.e. stability and instability of the wadi terraces, are reflected in the preserved, albeit relic paleosol horizons. The study describes and classifies the buried soils, place them within a stratigraphic framework, and evaluates their significance as proxies of the Holocene Humid Period as well as early human–environment interactions in Southern Arabia.

**Supplementary Information:**

The online version contains supplementary material available at 10.1038/s41598-026-55740-1.

## Introduction

Fluvial landscapes are increasingly under pressure from human exploitation and climate change. Due to their climate sensitivity and dynamic nature, alluvial plains in particular serve as important archives of landscape change and adaptation to shifting environmental conditions during the Quaternary period^1–3^. The alternation between sedimentation and soil formation is of particular interest in arid regions. The presence of buried soil sequences raises questions regarding the relationship between syngenetic and epigenetic soil formation during the weathering of the parent material, as can be observed, for example, in southern Italy^4^. As studies from the Negev in Israel show, soil formation is possible in active floodplains of the Holocene and Pleistocene, with the soils reflecting a complex interplay of sedimentation, erosion, and deposition^5^. Depth distribution of slightly soluble salts, calcium carbonate and gypsum precipitations indicates fluctuating water conditions, but also phases of stable alluvial surfaces^6^. Repeated phases of landscape stability and initial soil formation in Jordan during the Pleistocene suggest changes in hydrological conditions, which manifested as incipient erosion processes that may have prevailed throughout most of the Holocene^7^. Zielhofer et al. assumed that buried soils within alluvial sequences represent former stable floodplain areas, with alluvial soil formation corresponding to periods of reduced flood activity and low sedimentation rates^8,9^. Their examples from Tunisia and other regions in the Mediterranean have proven valuable for chronostratigraphic correlation across discontinuous alluvial facies. They also suggested that the duration of soil formation could be estimated using soil development indices. However, comparable stratigraphic and pedological data from Southern Arabia, and especially from Central Oman, remain scarce. In particular, the occurrence, properties and stratigraphic significance of in situ buried soils from the Holocene Humid Period (HHP) in this region remain poorly documented.

Based on a stratigraphically supported approach to regional floodplain dynamics, Faust and Wolf identified three main periods of alluvial soil formation in the Mediterranean region^10^: one at the end of the Pleistocene and two during the Holocene—from 7 to 5 ka (with a temporal gap from 6.5 to 5.5 ka), and between 3 and 2 ka. These soil forming phases were interrupted and framed by phases of fluvial activity, including channel aggradation, floodplain deposition, floodplain erosion and/or river incision.

Copeland et al. established a stratigraphy of Holocene alluvial soil units in the Sonora, Mexico^11^. The paleopedological record at La Playa revealed two different pedogenetic phases: one spanning from the Late Pleistocene to the Middle Holocene (> 15,000–4000 cal BP) and one for the Late Holocene (< 4000 cal BP). These two distinct phases of soil formation did not reflect contrasting climatic conditions, but rather extended periods of landscape stability^12^. Nevertheless, the human–environment interaction in the Quaternary worldwide was very dynamic, and the increase in the settlements was driven by favourable environmental conditions, such as around 4 ka^13^. At least temporarily, human occupation of wadi terraces appears to have been linked to arid climatic phases^14^. However, Völkel et al., emphasise the importance of climatic changes and demonstrate how extreme climatic events shaped both the landscape and ancient settlement patterns through aeolian and fluvial sedimentation^15^, making an understanding of anthropogenic influences on soils indispensable when interpreting soil-forming factors such as climate, organisms, relief, source material, and the duration of land surface exposure^16,17^.

In the United Arab Emirates^2,18,19^ and in Yemen^12^ phases of Holocene alluvial deposition are well documented. In the alluvial plains in the foreland of northern Jebel Hafit (UAE), sedimentation rates varied regionally, and fluvial deposition generally slowed after 10 ka. In the last two centuries alone, gravel deposition has reached up to 1.3 m^18^. From Oman, Woor et al. and Beuzen-Waller et al. provide information on alluvial deposits associated with reactivated river channels during the HHP^21,22^. Studies of the Hadramaut river system in Yemen showed that fluvial dynamics influenced Neolithic and Early Bronze Age settlement patterns in lowland areas, with periods of floodplain stability favouring settlement^20^.

A prolonged arid phase with reduced fluvial activity, characterised by desert pavement formation on stable surfaces, is recorded in Hadramaut from around 5200 cal BP^23,24^. Comparable observations at the desert margin in Yemen suggested that soil formation (with the exception of secondary calcareous soils and Arenosols) largely ceased after 6500 cal BP. Pedostratigraphic positions, AMS ^14^C ages of soil organic matter from paleosol horizons and specific soil properties such as organic matter enrichment suggest that in situ soil formation in alluvial areas was mainly confined to the HHP^25–27^. Isotopic data assign the HHP in Southern Arabia to 11.5 to 6.5 ka^28,29^, with the Early Holocene lasting only until 8.2 ka ago^30^. During this humid period, intense monsoon activity extended into central and northern Oman, while from 6.5 to 3.5 ka, precipitation decreased as aridity increased^31,32^. During this period, sediments began to form either through aeolian processes or through runoff associated with surface sealing^33^.

In arid regions—which are characterised by pronounced fluctuations in fluvial and alluvial erosion and deposition as well as intense seasonal flooding—well-developed in situ paleosols generally appear to be rare. However, the present study of terrace and floodplain sediments in Central Oman revealed a different picture: several buried soils have been identified, some formed under natural conditions, others in proximity to archaeological sites. several buried soils were identified, some of which formed under natural conditions, others near archaeological sites. The description and analysis of natural buried soils as “reference soils” is therefore of crucial importance, not only for stratigraphic purposes but also as local indicators of the HHP in Southern Arabia. Buried soils including paleosol horizons serve as geoarchive for reconstructing the landscape prior to or concurrent with early human settlement^34–36^.

### Background and hypotheses

**Fig. 1:**
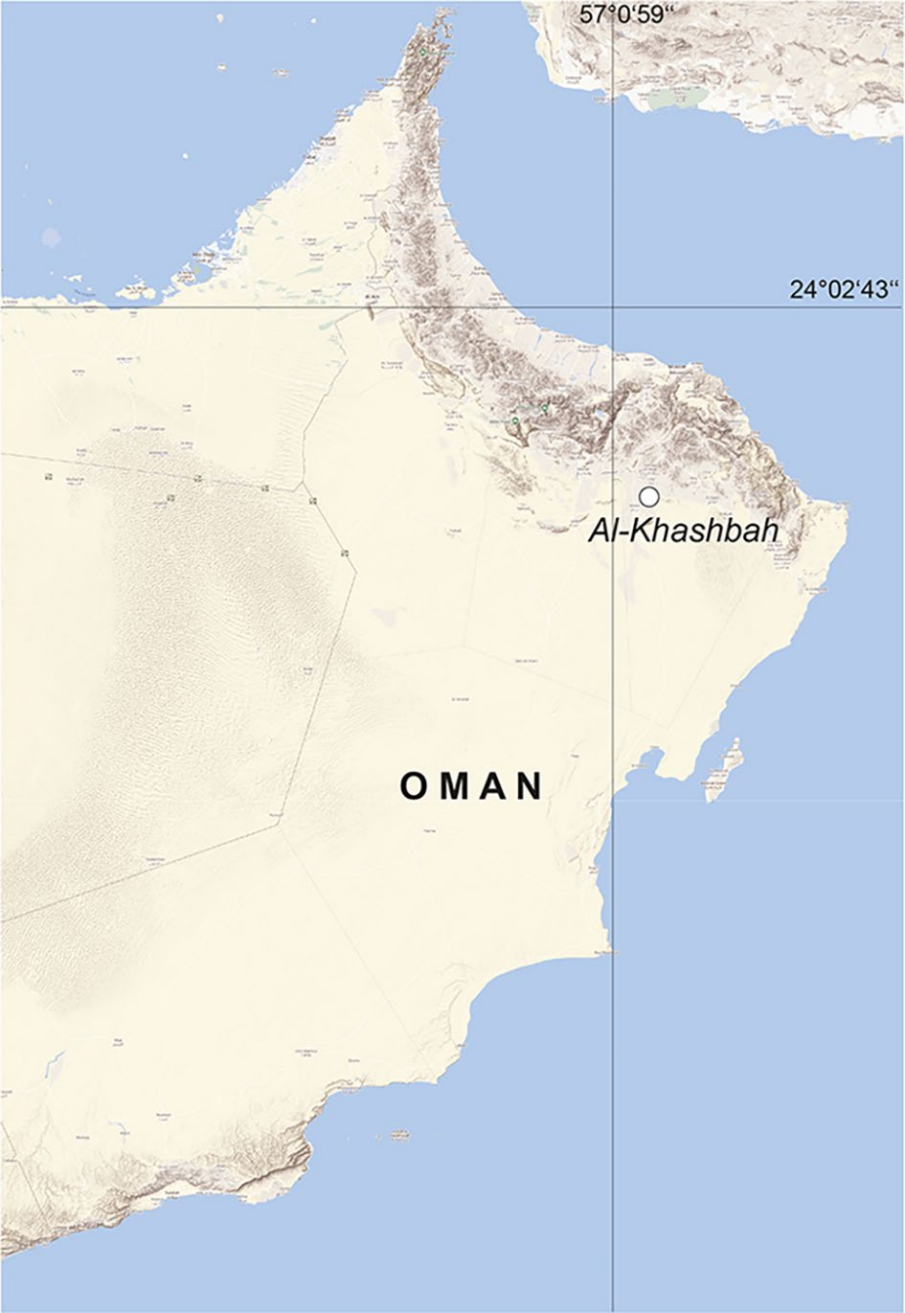
Location of the investigation area in the foreland of the Al-Hajar Mountains (created with Adobe Illustrator based on https://openstreetmap.maps.arcgis.com/apps/mapviewer/index.html?webmap=e409ec0a5ef94d5cb486571894143b7c).

As part of the BMBF project, sediments and alluvial soils in Wadi Shuwayhi in Al-Khashbah (Fig. 1), were studied in order to distinguish temporarily stable surfaces from geomorphodynamically ones and to assign them to specific alluvial phases and climatic shifts. Another objective was to investigate the human–environment relationship during the Early Bronze Age through interdisciplinary analyses of paleoecological archives such as sediments and soils, pollen, charcoal, and gastropods^37^. Al-Khashbah was selected due to its numerous Early Bronze Age archaeological structures and its wadi system. Arabian wadi systems are characterised by high-energy hydrological regimes and severe seasonal flooding, which produce broad, branched, and unstable river systems. Lateral erosion undermines wadi banks and forms erosion terraces, causing the wadi bed and associated alluvial formations to widen progressively. These dynamic processes pose significant challenges to the preservation of paleosols and other environmental archives near active river channels.

This study introduces an “undercover proxy” and also provides the first evidence of paleosols located within a fluvial terrace system of a low-elevation alluvial plain in Central Oman. As noted above, it is difficult to distinguish individual phases of soil formation in the context of fluctuating river erosion and deposition processes. Accordingly, this study tests the following hypotheses:

#### H1

Buried soils that formed under Holocene humid conditions have been preserved within alluvial deposits.

#### H2

 Human activities, including cultivation, altered the alluvial deposits at Al-Khashbah during the Early Bronze Age.

#### H3

The stages of soil formation coincide with periods of reduced river activity, characterised by a narrowing of the floodplain, overbank sediment deposition, limited channel shifting, and stabilisation of the wadi banks.

Based on these assumptions our study aims to (i) describe and classify the buried soils, (ii) place them within a stratigraphic framework, and (iii) assess their significance as indicators of the HHP and early human–environment interactions in Arabia.

### Al-Khashbah and its environment

**Fig. 2:**
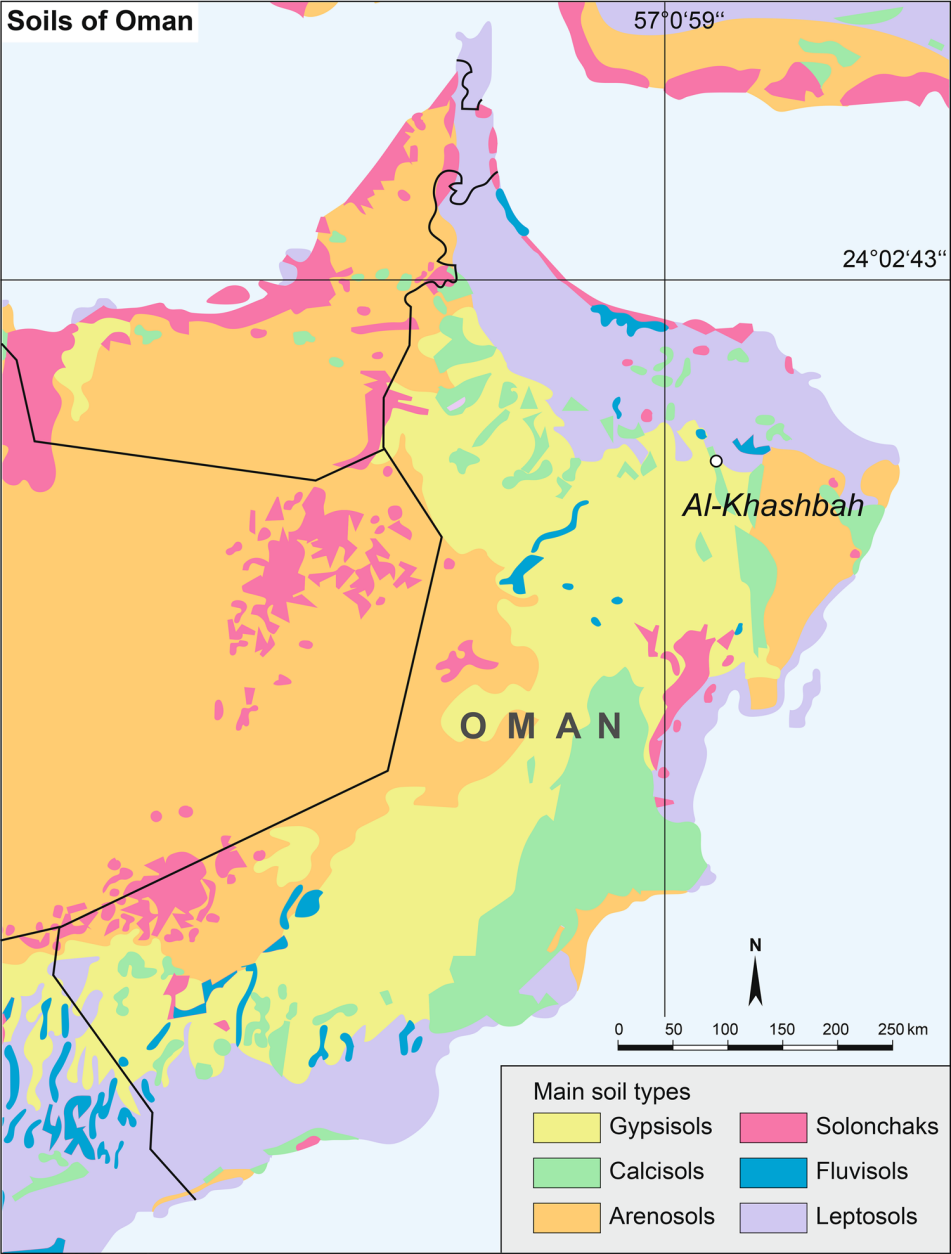
Modern soil distribution in Oman (created with Adobe Illustrator based on https://esdac.jrc.ec.europa.eu/public_path//shared_folder/Atlases/Soil%20Atlas%20Asia_online_Part_1.pdf, p. 76).

Al-Khashbah, located 14 km northwest of the district capital Al-Mudhaybi, is one of the largest Early Bronze Age sites in Central Oman^37^. The surveyed area extends 5 km east–west and 3 km north–south and is situated in the floodplain of Wadi Samad at its confluence with Wadi Shuwayhi. The watershed has a size of about 444 km^2^ with main tributaries reaching the Al-Hajar Mountains in the north (Fig. 1). The landscape is dominated by an extended alluvial plain and limestone outcrops organised in several ridges running from west to east. Wadi Samad and Wadi Shuwayhi originate in the Samail ophiolite complex of the Al-Hajar Mountains. The headwaters are dominated by harzburgite, gabbro and peridotites^38^. The aeolian silty-sandy sediments (khabrah) cover large parts of the alluvial plain. Wadi Samad and Wadi Shuwayhi are nowadays ephemeral streams that flow only during the annual rainfall events (with high interannual variability). The current climate corresponds to that of an extremely arid subtropical desert, with typical annual precipitation of approximately 80 mm and vegetation characteristic of the drought-affected *Euphorbia larica-Acacia tortilis* woodland type, which extends across the entire southern foothills of the Al-Hajar Mountains. The Soil Atlas of Asia (Fig. 2) describes the soil regimes of Central Oman as thermic soils with a mean annual temperature of ≥ 15 °C but below 22 °C, a difference between mean summer and winter soil temperatures of ≥ 5 °C, and a widespread aridic soil moisture.

Modern soils in Central Oman are Calcisols, Gypsisols and Fluvisols, which develop in terrace and floodplain sediments (Fig. 2). Solonchaks are found in depressions and oases, and very shallow Leptosols occur along outcrops and in the mountains. Regosols of undifferentiated fine and medium texture are very widespread in various morphological settings^39^. Dust from eroded Gypsisols in southern and Central Oman is a local and regional source of recent gypsum precipitation in the sediments covering the paleosols.

### Sedimentary facies of terrace fills

**Fig. 3:**
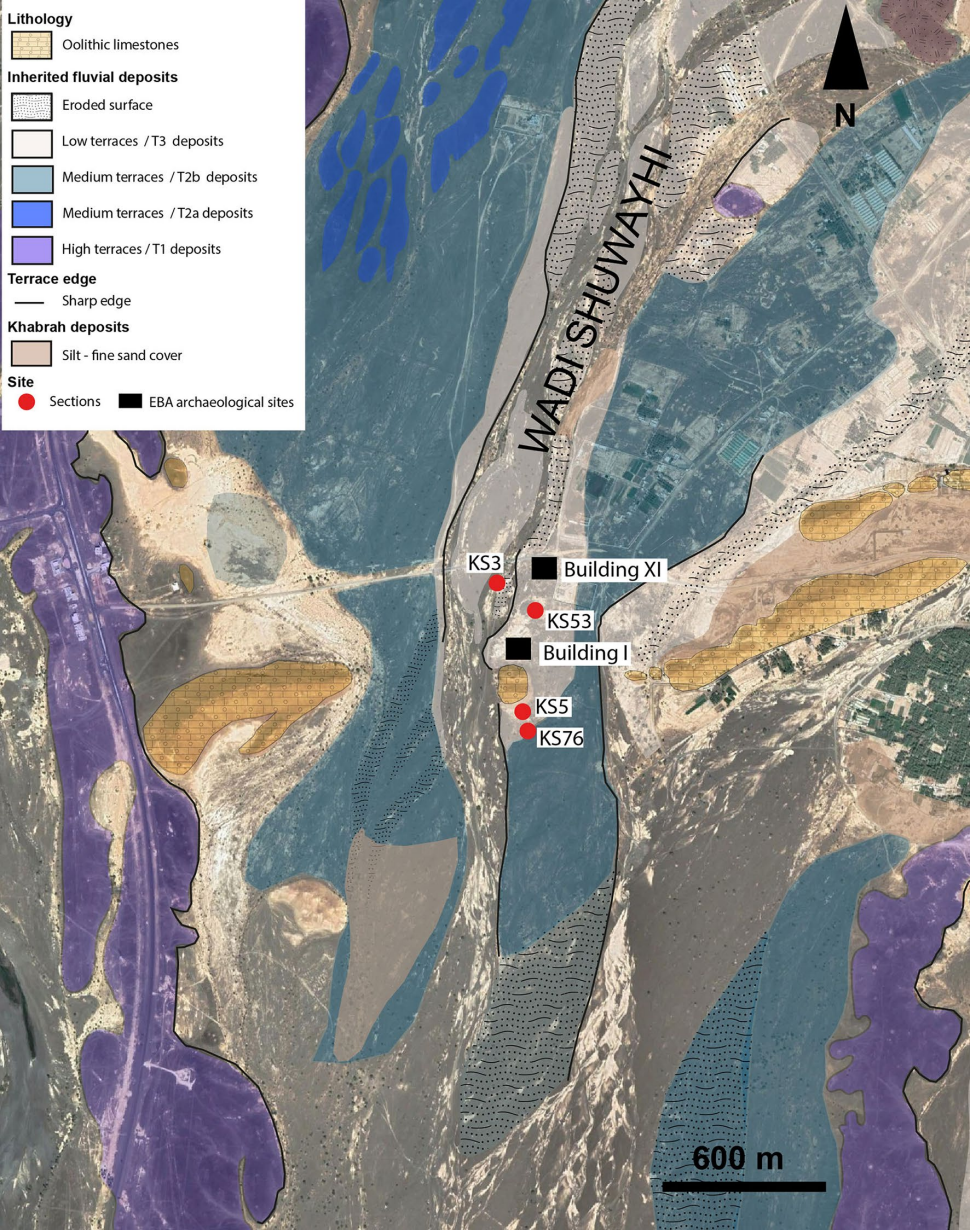
Geomorphological map with sections and archaeological sites (created with Adobe Illustrator based on https://www.google.de/intl/de_ALL/earth/versions/#earth-pro, 2021).

The alluvial plain of Al-Khashbah features several levels of fluvial terraces (fill terraces), which are classified from T1 to T3 based on their elevation (T1 the highest, with + 10/15 m above wadi bed; T2 with 2/2,5 m above wadi bed and T3 1/1.5 m above wadi bed). The medium to low terraces occur mostly near the present floodplain of Wadi Samad and Wadi Shuwayhi (Fig. 3), particularly the lower terrace level, which indicates Holocene deposition, similar to other low-level terrace systems that have been described and dated in Oman^21,22,40^. In Southern Arabia an Early Holocene from 11.5 to 8.1 ka is well known, which is associated with an initial Holocene sedimentation phase. The second aggradation phase has been assigned to a period of 7.0–5.1 ka, and after 5100 BP accumulation decreases due to reduced runoff/precipitation. The reason for this is the southward migration of the ITCZ^23^. The current erosion of the wadi bed extends in some places down to the bedrock or conglomerates. At present, the wadi floor is heavily disturbed by gravel extraction, which locally intensifies erosion.

On the lower terrace level in the Al-Khashbah area, the following fluvial sedimentary facies can be regularly observed:

1. Gravel- and sand-dominated deposits from the main channel, comprising well-rounded pebbles either well-sorted or

Heterometric in size. Debris flow-like deposits also occur.

2. Fine-grained sediments, ranging from silty fine sand to sand, interpreted as the result of suspension load deposition during low-energy flood events or in the distal part of the floodplain (overbank deposits).

In some places, redox-induced transformations overlie the sedimentary structure, resulting in mottled gray-blue to greenish colours, sometimes accompanied by ochre-red to black iron and manganese coatings and linings. The finer, silty-sandy sediments are associated with the deposition of suspended sediments during low-energy flood events or with deposits in the distal part of the alluvial plain. The gravel-dominated facies is interpreted as comprising both larger and smaller channel fillings as well as sandbar surfaces, reflecting the dynamics of sediment transport and channel remodeling during periods of high discharge. The main sediment sources are the weathered ophiolites from the Al-Hajjar Mountains as well as wind-blown sediments from the desert and the plains.^2^.

## Methods

### Field methods

The four sites were selected as part of the geomorphological mapping of Wadi Shuwayhi. All profiles on the lower terrace showed signs of past soil formation (humus patches, bioturbation, aggregation). One of the buried horizons (KS3) exhibit traces of human impact, and its upper buried soil can be seen as a comparative horizon for diagnostic paleosol horizons. Altitude and position of the section were using a DGPS with RTK mode. The profiles were dug, cleaned and leveled.

The description of the layers (L1 to L11/L17/L18, bottom-top) was based on several criteria: position of the layer, general structure (loose, cemented, laminated, massive, etc.), texture, colour, visible results of soil-forming processes (particularly CaCO_3_ precipitation, iron oxidation, redox-induced patterns), presence or absence of roots/rhizoliths and other organic remains (charcoal, gastropods, C_org_). The soil horizons were determined in accordance with the WRB^39^. After the description, the sections were drawn by hand and then digitised. The strategy for sampling bulk samples and for radiocarbon dating was carried out either in depth increments of 5 or 10 cm and/or horizontally. 5 cm sampling was used for palynological investigations, which have be carried out in the project, conducted as part of the project but is not relevant to our approach. For reasons of efficiency sample sharing was performed when appropriate. In addition, three micromorphological samples were collected from KS3 using Kubiëna boxes, and three fossil shells of adult specimens of the terrestrial snail *Zootecus insularis* were selected for δ^13^C and δ^18^O measurements. OSL sampling in selected layers was performed using steel tubes taking care to avoid direct sunlight.

### Laboratory methods

Bulk samples were air-dried and then sieved using a 2 mm mesh. All samples were analyzed for electrical conductivity (slightly soluble salts), pH, total carbon (organic and inorganic), and grain size. C and S mass% contents were determined by oxidative heat combustion at 1150 °C in a He-atmosphere (element analyser ‘vario EL III’, Elementar Analysesysteme GmbH, Germany, in CNS mode). Soil organic C content (C_org_) was calculated using the formular C_org_ = C_total_ − CaCO_3_ × 0.12. CaCO_3_ contents were measured volumetrically by CO_2_ evolution using a calcimeter (‘Calcimeter’, Eijkelkamp, Giesbeek). Grain size was determined by laser diffraction using a Malvern Mastersizer 2000 laser particle size analyser. Sediments were treated with hydrogen peroxide (not with HCl) and the sediment-suspended in sodium hexametaphosphate solution. The percentage of coarse elements was measured by weighing (total sample weight and weight of elements < 2 mm in diameter).

Major and trace elements were analysed using a wavelength dispersive XRF device^41^. First the samples were ground in an agate mill for 10 min. Loss on ignition was determined at 1000 °C. Measurements on fused beads were performed on 32 standardised samples using a Bruker AXS S4 Pioneer XRF device (Rh-tube at 4 kW). The international standards used are compiled in Govindaraju et al.^42^. The Chemical Index of Alteration (CIA) after Nesbitt and Young was calculated^43^. CIA = [Al_2_O_3_/(Al_2_O_3_ + CaO* + Na_2_O + K_2_O)] × 100, while CaO* is the amount of silicate-bound CaO. The CaCO_3_ was calculated out beforehand. The index is often used to estimate the conversion of feldspars into clay minerals: the higher the index, the more advanced the weathering (< 50 slightly weathered). After impregnating the air-dried micromorphology samples with Oldopal P 80–21, the hardened blocks were cut and sliced into 90 × 60 mm-thin sections and scanned with a flatbed scanner. The thin sections were described at 50–200 magnification under a polarizing light microscope (Zeiss Imager.A2; Software AxioVision 4.7.2), mainly using the terminology of Stoops^44^. *Zootecus insularis* shells were freed from dirt and debris by mechanical cleaning, then placed in Eppendorf cylinders with deionized water and shaken for 24 h on a shaking table. The specimens were subsequently subjected to repeated ultrasonic rinsing before being dried in an oven at 40 °C for 24 h. The samples were then analysed using Raman spectrometry to preclude the presence of any diagenetic alteration before carbonate powders were collected for δ^13^C and δ^18^O measurements. Depending on the age and the correlated size of the animal a various number of samples was taken per shell (Fig. 9). Further data of 104 *Zootecus insularis* shells from all sections in Al-Khashbah were linked to five specific environmental settings on the basis of their δ^18^O_shell_ pattern and compared to other climate proxies (Fig. 10). For further description see Schmitt et al.^45^. The powder of shells was sent to the Curt-Engelhorn-Zentrum Archäometrie gGmbH in Mannheim for AMS ^14^C dating^46^, and bulk soil-samples have been analysed in the AMS lab in Poznan (suppl. Tab. 2). After removing limestone and soluble humic acids, samples were graphitised and converted to gaseous CO_2_ via combustion, and then reduced to elemental carbon with hydrogen at 550 °C. Calibration was performed in Oxcal 4.4^47^ using the latest calibration dataset IntCal20^48^. OSL was applied at the Baylor University, College of Arts and Sciences, Department of Geosciences: Quartz extracts of a particular particle size range (63–100 µm) were isolated for single aliquot regeneration (SAR) protocols^49^. SAR protocols were completed using an Automated Risø TL/OSL–DA–15 system. The grains were subjected to optical stimulation at 125 °C with a heating rate of 5 °C/s. All SAR emissions were integrated for the first 0.8 s out of 40 s of stimulation, with background emissions at 30–40 s. The fast ratio was calculated for each natural material and the equivalent. An assessment of the environmental dose rate for each sample was estimated based on sediment volume using inductively coupled plasma mass spectrometry (ALS Laboratories, Reno, NV). A cosmic ray component, considering location, elevation and depth of strata sampled was calculated which includes the soft component^50,51^. The moisture content (by weight) for the burial period was estimated from current values, sedimentology and with reference to field indicators on the height of the water table. The “Luminescence Dose and Age Calculation” (LDAC) platform resolved a final OSL age with corresponding statistical and error analyses^51^. All laboratory data can be found in the supplementary data^37^.

## Results

### Sediments and soils in Wadi Shuwayhi

**Fig. 4:**
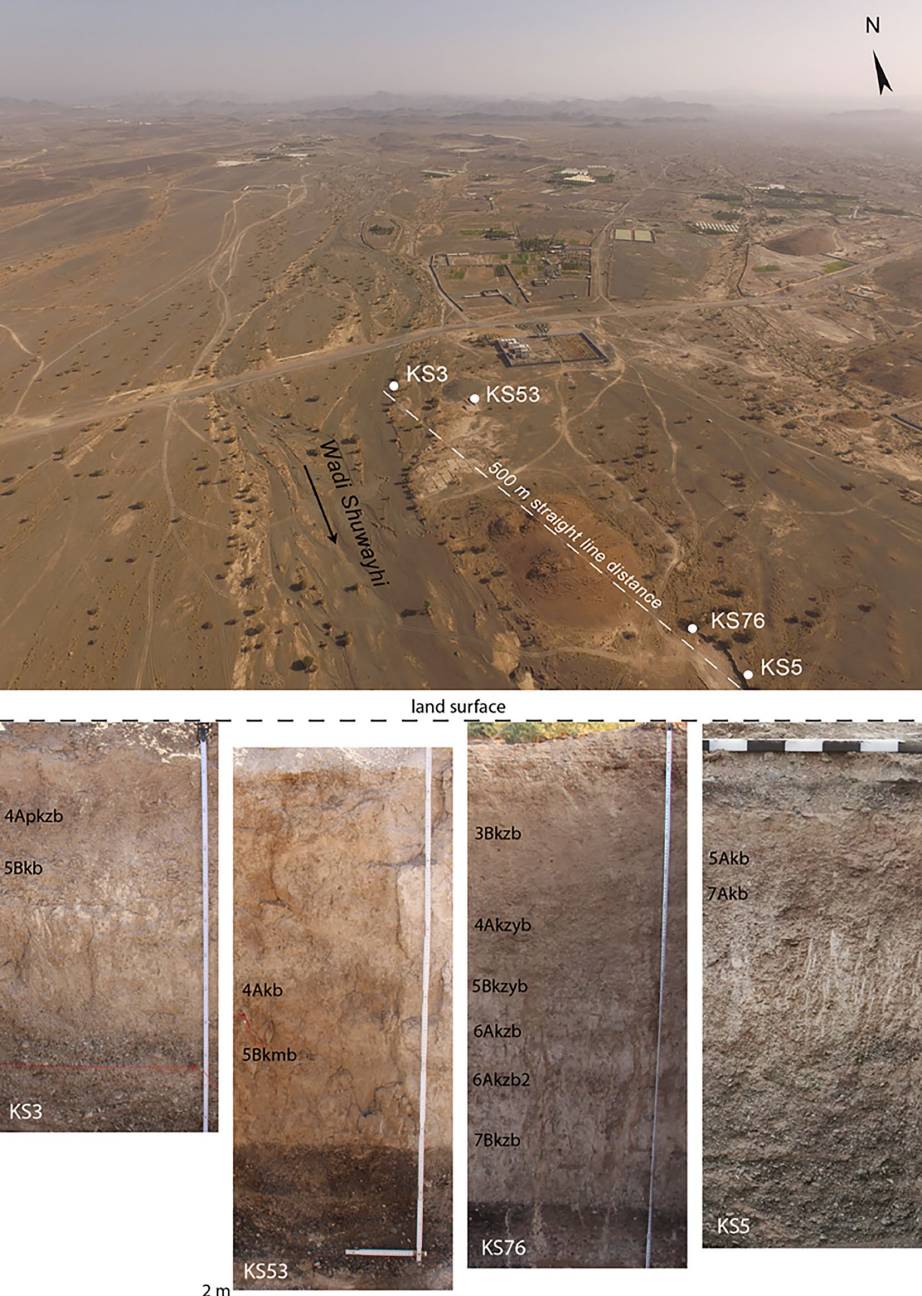
Upper photograph—view from the south to the foothills of the Hajar Mountains; lower photographs—soil profiles including paleosol horizon designation.

The profiles KS3, KS53, KS76 and KS5, located on the left bank of Wadi Shuwayhi (Fig. 4) are dominated by fluvial main-channel and overbank deposits. Runoff sediments mixed with pedosediments (i.e. reworked and transported paleosol material) are also part of the lower terrace. Three main sedimentary units with distinct stratigraphies can be distinguished in the profiles:

1. Major or minor channel fillings at the base, consisting of gravel- and sand-dominated deposits, often with horizontal stratification.

2. Transitional facies from coarse fluvial deposits to finer deposits, associated with low-energy depositional conditions; consisting of reworked eolian silt or material from the floodplain, occasionally enriched with coarse-grained material a) with calcrete development, b) without calcrete development.

3. Overbank deposits partially covered by aeolian (khabra) or reworked aeolian sediments; often with a desert pavement on the surface.

The paleosol horizons in all four profiles share the following properties: soil formation is largely limited to aggregation, bioturbation (caused by roots and animals) and a slight accumulation of soil organic matter. Secondary calcification occurs in all profiles, gypsum precipitation and the enrichment of slightly soluble salts occur only in KS3 and KS76 (Fig. 6 and suppl. Tab. 1). It should be noted that CaCO₃ precipitation, at approximately 15–50%, has altered the original sediment properties. Fossil gastropod shells from the Early Holocene are present in some sections. As done with micromorphology, KS3 was selected to present isotope data: snail shells were analysed to assess their suitability for stratigraphic and climate change-related interpretations.

### Soil profile description

#### KS3 *Salic Calcic Anthric Fluvisol*

Profile KS3 is located on the lower terrace of the left bank of Wadi Shuwayhi (E 605,082,95, N 2,506,602,66).

South of this section lies the Early Bronze age building XI (Figs. 3 and 4). The site is currently affected by lateral erosion but shows no signs of recent human activity. It is assumed that the area was used for agriculture during the Bronze Age, whereas today it is subject to intensive grazing.

##### 4Apkzb horizon (L8)

The upper buried soil horizon consists of fine overbank deposits. It exhibits a crumb structure, a brownish colour, and contains rhizoliths and root channels. Compared to the underlying layer, the soil matrix appears disturbed and heterogeneous. The upper boundary is clear, horizontal and wavy, the lower boundary of this horizon sharp and straight. Radiocarbon dating of soil organic matter yields an age of 4817–4450 cal BP (2868–2501 cal BC), while OSL dating of the sediment yields a deposition age of 8065 ± 225 a.

##### 5Bkb horizon (L7)

The paleosol horizon below formed within an overbank deposit with an upper section composed of a coarser matrix. The soil exhibits an angular to subangular, blocky structure. Its silt content is slightly higher than that of the overlying unit (L9-L11), indicating an increased proportion of fine carbonates—the colour is correspondingly paler. CaCO_3_ precipitations occur as small carbonate nodules and as fragments of a cemented crust. The lower boundary of this horizon is clear, horizontal and straight. Radiocarbon dating of soil organic carbon yields an age of 8689–7961 cal BP (6740–6012 cal BC).

##### Stratigraphic interpretation

**Fig. 5:**
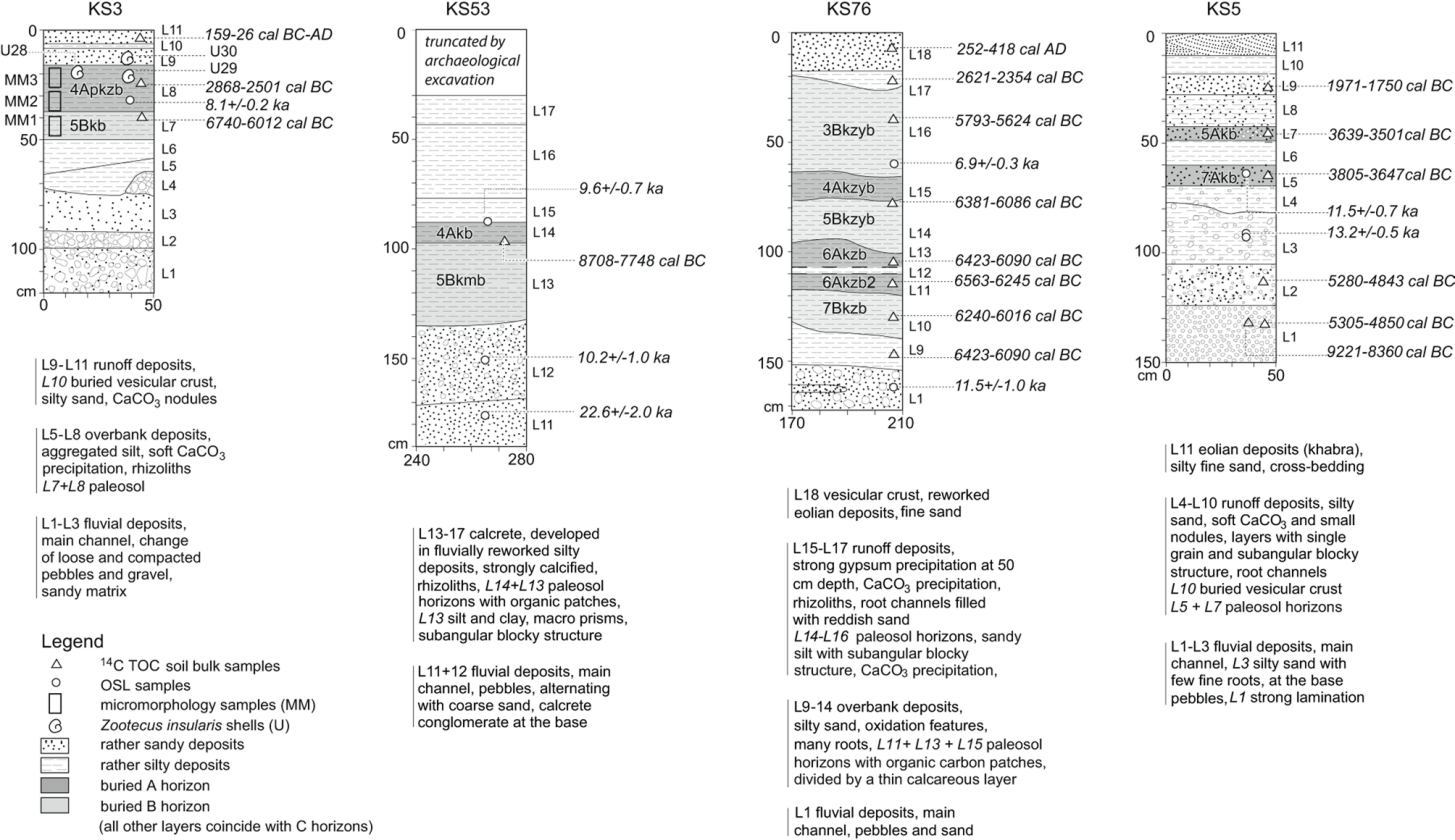
Soil profiles (numbers of sediment layers follow bottom to top field description, for C horizons Table 1; description of layers is summarised in layer units (L1-L3 etc.); sediments belong to either runoff, overbank or fluvial deposits/main channel; micromorphology Figs. 7 and 8, radiocarbon ages of snail shells Fig. 9, all AMS and OSL data suppl. Tab. 2).

Three sedimentary units were identified from top to bottom (Fig. 5): runoff deposits—overbank deposits—fluvial deposits (main channel). The buried horizon 4Apkzb, dated to 4800–4400 cal BP, formed in a sediment dated to 8.1 ka. This sediment layer (L8) covers the in situ buried paleosol horizon 5Bkb which is dated to 8700–7900 cal BP. The absence of an 5A horizon could be explained by erosion during a phase of increased fluvial activity. The 4Apkzb horizon with a significantly higher C_org_ content is likely a result of agricultural use. The apparently sharply defined lower boundary of this buried anthric horizon (not a HHP paleosol) could be the result of continuous digging.

##### Laboratory data

**Fig. 6:**
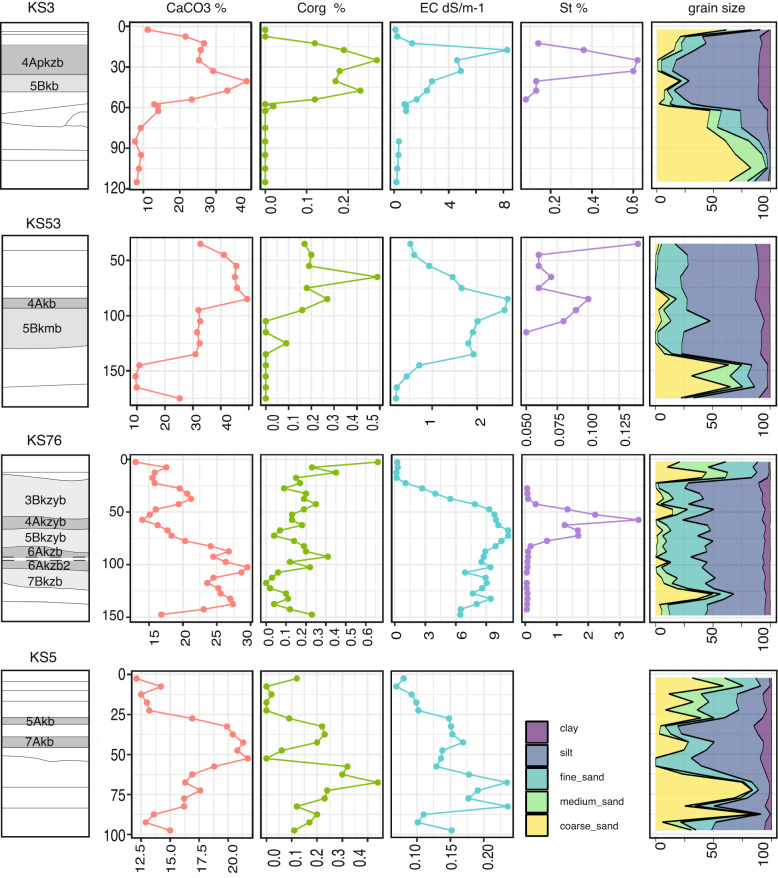
Diagrams showing the depth distribution of diagnostic soil features (created with R 4.5.3); for all lab data suppl. Tab. 1

The depth function of coarse sand, fine sand, and silt clearly show the layering of the KS3 deposit (Fig. 6 and suppl. Tab1). The coarse-grained fractions are associated with deposits in fluvial main channel (L1 to L3), whereas the upper layers (5 to 8) are dominated by silt and associated with overbank deposits. Silt contents range between 60 and 75%, including proportional amounts of carbonates. The soil is heavily salt-enriched, with EC values exceeding 8 dS m^-1^.

Soil pH values range from 8 to 8.5, indicating alkaline conditions, while the fluvial sediments at the base of the sequence are extremely alkaline (pH 9–10). The organic carbon content of the buried soil ranges between 0.20% and 0.25%. CaCO_3_ content varies between 25 and 40%, with lower values in the A horizon than in the B horizon. CIA values of the buried soils range from 46 to 50, indicating low weathering intensity (< 50), and slightly increase with depth.

#### Micromorphology of KS3

##### 4Apkzb horizon (L8)

The entire layer shows clear signs of reworking by soil mesofauna (abundant passage features with loose crumbly, mostly continuous infillings) and alteration by human activity. The crystallitic b-fabric exhibits a high concentration of micritic carbonate particles (< 5 µm) within the micromass, which displays a dark brown to grey speckled limpidity. The coarse -grained material consists of limestone fragments, alterites (weathered mineral fragments retaining the original fabric, here mainly olivine), sparitic nodules and shell fragments.

**Fig. 7:**
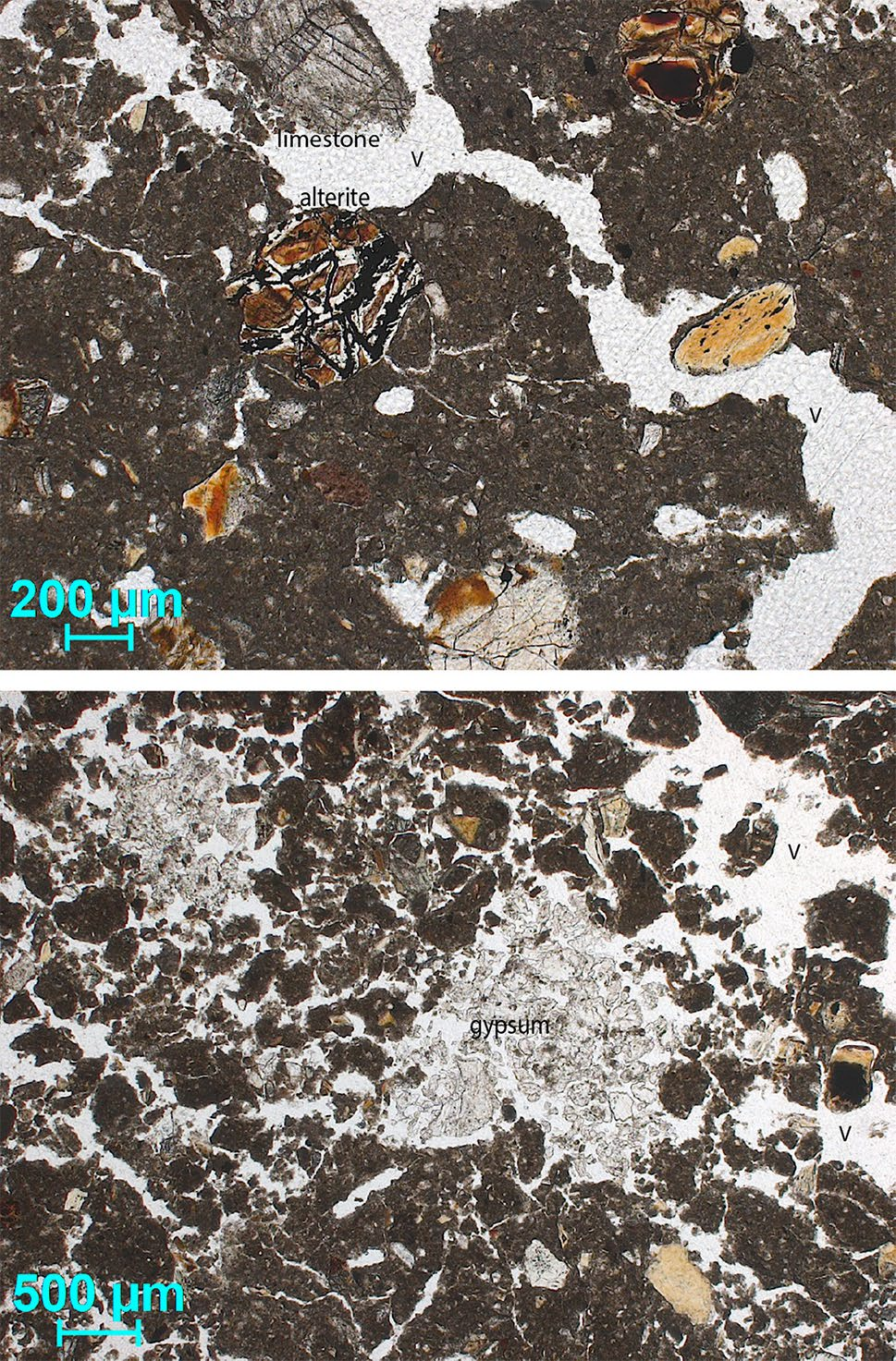
Micromorphological features of KS3 anthric horizon 4Apkzb(L8) upper photograph—plane polarised light: subangular blocky microstructure, voids (v); coarse material consists of fresh limestone fragments, alterites and rounded bone fragments; dark grey areas represent micritic calcium carbonate masses; lower photograph—plane polarised light: crumb micro-structure; speckled light grey areas are pedogenic gypsum precipitations.

In the upper part of the 4Apkzb horizon a subangular blocky to crumb microstructure with a well to moderately developed pedality is observed, with no gypsum precipitations present. The coarse material contains rounded, elongated bone fragments up to 300 µm in diameter) (Fig. 7 upper photograph), along with micritic nodules. In the lower part, a crumb to compacted single grain microstructure with a well-developed pedality and a high degree of separation is present.

Tabular gypsum infillings occur in channels and voids (Fig. 7 lower photograph), accompanied by root or nutshell residues. Fragments of micritic nodules are present in the groundmass. The gypsum precipitation in voids suggests that they were introduced by irrigation water, followed by capillary evaporation. The differing microstructures in the upper and lower parts indicate different types or intensities of agricultural activity.

##### 5Bkb horizon (L7)

**Fig. 8:**
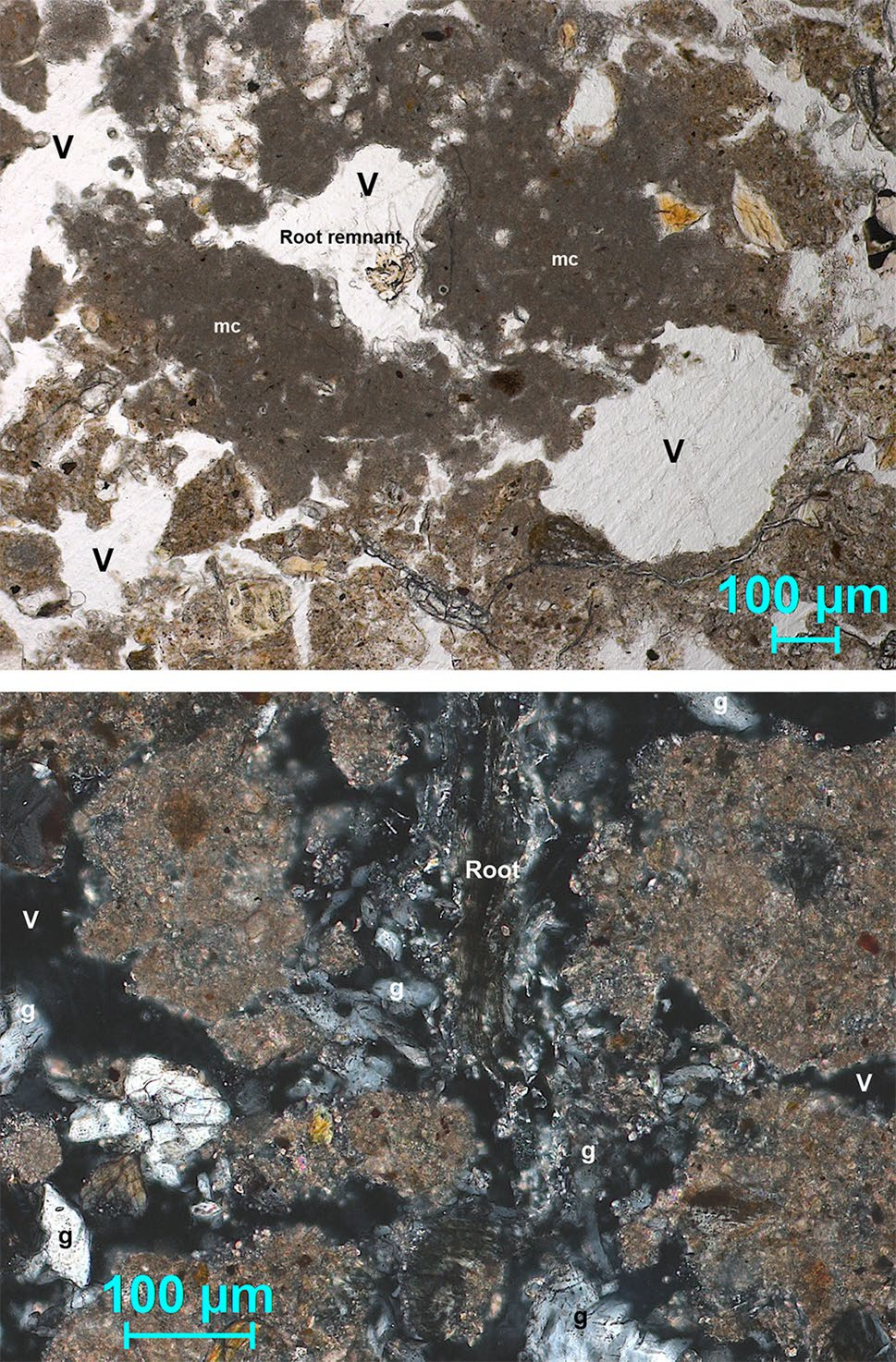
Micromorphological features of KS3 paleosol horizon 5Bkb (L7) upper photograph—plane polarised light: subangular blocky crumb microstructure, dark grey areas represent micritic carbonate precipitations and hypocoatings (mc) around voids; lower photograph—crossed polarised light: subangular blocky to crumb microstructure with crystallitic b-fabric in peds; root residues in voids (v) with associated tabular to lenticular gypsum precipitations (g).

The predominant micromorphological properties are subangular blocky to crumb microstructures (Fig. 8 upper photograph) with moderately pronounced pedality and a crystallitic b-fabric with a brown speckled limpidity of the micromass. Frequently occurring micritic hypocoatings represent secondary calcium carbonate precipitations. Voids and channels contain abundant calcified root residues as well as lenticular and tabular gypsum precipitations (g) (Fig. 8 lower photograph). Secondary carbonate and gypsum formed through downward percolation of (irrigation) water and subsequent capillary evaporation. Compared to layer 8, the micromass exhibits porosity, while the coarse-grained material contains a higher proportion of micritic nodules. Vertically oriented root residues indicate a horizon that has remained unaffected by agricultural activities. This buried paleosol horizon represents the relic of an in situ developed soil.

#### KS53 *Petrocalcic Calcisol*

Profile KS53 is part of a trench excavated in a north–south direction along the southern edge of Building XI (E 605,151,01, N 2,506,526,79; cf. Figure 3). The soil profile, consisting of layers L11-L17, is located directly adjacent to the outer ditch of the archaeological trench. In L13 and L14, a two-layered soil is preserved beneath a massive calcrete. At the transition to layer 15, root channels and humus-rich patches can be observed over a long distance, possibly indicating former patches of vegetation. The area is used for extensive grazing.

##### 4Akb (L14)

This shallow soil formed in reworked silt, most probably representing an overbank deposit with a high amount of aeolian material (khabra). Coarse material is nearly absent. Soil aggregates occur as subangular blocks, and CaCO_3_ precipitation is visible in various forms (carbonate powder, nodules, calcified rhizoliths). The colour is pale brown except for the dark-grey humic patches. The upper boundary is clear, horizontal and straight, the lower boundary of this horizon is diffuse and patchy. Radiocarbon dating of the organic matter (from patches) yields an age of 10,657–9697 cal BP (8708–7748 cal BC).

##### 5Bkmb (L13)

The parent material of this soil horizon consists of a silty-sandy overbank deposit containing up to 20% coarse material. The aggregates are fairly well-developed subangular to angular blocks. An initial development of a columnar structure can be observed. This horizon is slightly cemented, and CaCO_3_ precipitation occurs in various forms similar to those in the overlying horizon. The colour is light brown, and ancient root channels as well as rhizoliths are present. However, in the surrounding material no C_org_ is detectable. The lower boundary of this horizon is sharp, inclined and wavy.

##### Stratigraphic interpretation

From top to bottom, two sedimentary units can be distinguished (Fig. 5): (reworked) overbank deposits and main-channel deposits. The paleosol horizon 4Akb, which contains humic root patches, likely formed in reworked aeolian material or within a large-scale nabkhah. The underlying 5Bkm horizon is cemented and lacks soil organic matter. Both the A and B horizons occur between fluvial deposits and a massive calcrete. The paleosol, whose formation started after 10 ka, marks the beginning of a patchy vegetation distribution. Soil formation likely proceeded parallel with sediment deposition. OSL dating of the sediment (L12) beneath the soil yields a deposition age of 10,320 ± 1085 a; the sediment of L15 yields a deposition age of 8065 ± 225 a. The paleosol horizon 4Akb with a radiocarbon age of 10,657–9697 cal BP could represent a suitable natural “reference soil” for the onset of the HHP. Although it is located next to a ditch, the soil profile itself shows no signs of human activity.

##### Laboratory data

The depth distributions of coarse sand, fine sand, and silt clearly show the stratification of the profile. The coarse sand fractions are associated with fluvial main-channel deposits (L11 and L12), while the upper layers (L13-L17) are dominated by silt and fine sand. The silt content ranges between 60 to 70%. The soil is slightly salt-affected, with EC values between 2 and 2.5 dS m^-1^ (Tab. 1). Soil pH values are around 8.5, indicating alkaline conditions, while the underlying fluvial sediments are extremely alkaline (pH ~ 9). Sulphur, present as calcium sulphate (gypsum), is negligible. No organic carbon is detectable in the matrix of the buried soils, with the exception of material derived from organic matter of former root channels in the 4Akb horizon. The CaCO_3_ content is approximately 30%, with the underlying river sands (with the exception of the calcareous crust) containing lower amounts. The CIA values of the buried soil are approximately 75, indicating a comparatively high weathering intensity (> 50), i.e., the transformation of feldspars into clay minerals^43^.

#### KS76 *Salic Calcic Gypsic Fluvisol*

Profile KS76 is located north of KS5, near building XI (N 605,157,37, E 2,506,179,04; Fig. 3). The site is currently not affected by lateral erosion, but has been altered by technical excavation. The paleosurface is slightly inclined from NE to SW, as indicated by the dip of strata. At present, the area around the nabhkah wit a big *Acacia* is flat and used for extensive grazing. For pollen analysis, samples were collected in 5-cm increments, allowing for higher resolution and more samples for radiocarbon (TOC) dating.

##### 3Bkzyb horizon (L16)

The paleosol horizon 3Bkzyb formed in silty-sandy runoff sediments, with clear signs of gypsum precipitation visible in the lower part and at the transition to L15. Gypsum-filled root channels and a „bowl “-shape structure (approximately 80 cm in diameter) extend downward beyond the horizon boundary. The material is loose, pale beige, and rich in gastropods. There is little angular gravel present. Two large root channels filled with reddish sand and iron oxide traverse this horizon and continue down to L8. An accumulation of organic soil matter and bioturbation features are observed. CaCO_3_ precipitation occurs both within root channels and finely distributed in the matrix. The upper and the lower boundaries are clear, inclined and wavy. The radiocarbon age (TOC) of the soil organic matter of the 3Bkzyb horizon is 7742–7573 cal BP (5793–5624 cal BC), while the sediment of this layer is dated to 6915 ± 345 a using OSL.

##### 4Akzyb horizon (L15)

This part of the soil also formed from silty-sandy runoff sediments, but here it exhibits a weak crumb structure caused by gypsum precipitation. Large CaCO_3_ nodules are present. Traces of bioturbation are visible throughout the horizon, and the root channels are filled with reddish sand. CaCO_3_ precipitation appears as calcified coatings around the root channels. The soil colour is pale brown. The upper and the lower boundaries are clear, inclined and wavy. The radiocarbon age of the soil organic matter in this horizon ranges from 8330–8035 cal BP (6381 to 6086 cal BC).

##### 5Bkzyb horizon (L14)

The fine sand and silt of the overbank sediment form a porous material. Within the horizon, there is an irregular, thick band of CaCO₃ deposits containing soft nodules and calcified rhizoliths. The aggregates are subangular and blocky, and bioturbation resulting from intense root growth has created an inclined and wavy lower boundary.

##### 6Akzb horizon (L13)

The buried paleosol horizon formed in silty overbank sediments. The upper boundary is irregular and disturbed; isolated pebbles are part of the matrix. A weak subangular blocky structure is present. CaCO_3_ is finely distributed but does not occur in the form of nodules. The colour of the horizon is brown. Most conspicuous are the humic spots distributed horizontally across the entire horizon. Numerous bioturbation features are present, and root channels show different root generations. The lower boundary is clear, horizontal and wedge-shaped. The radiocarbon age (TOC) of the humic patches is 8372–8039 cal BP (6423–6090 cal BC).

##### 6Akzb2 horizon (L11)

The buried paleosol horizon is genetically related to horizon 6Akzb. A thin calcareous layer (L12) containing soft nodules seperates this horizon from 6Akzb. The lower boundary was originally sharp and horizontal, but has been altered by deep roots (wedge-shaped). The radiocarbon age (TOC) of the humic patches is 8512–8194 cal BP (6563–6245 cal BC).

##### 7Bkzb horizon (L10)

Fine sand and silt form the overbank deposit. Soft CaCO_3_ nodules and calcified rhizoliths occur within the horizon. The aggregates are weakly subangular blocky, bioturbation features result mainly from root growth.

##### Stratigraphic interpretation

From top to bottom, four sedimentary units can be distinguished (Fig. 5): reworked aeolian deposits—runoff deposits—overbank deposits—fluvial main-channel deposits. Within this sedimentary sequence, three buried paleosol A-horizons are present. All of them formed between 8100–8400 cal BP. The 6Akzb and 6Akzb2 horizons with patches of humic material related to natural vegetation/root pattern, indicate an in situ development occurring concurrently with gradual sediment accumulation and weathering. The soils formed in L14 to L16, characterised by pronounced gypsum precipitation and “bowl shaped” lower boundaries, reflect drying processes associated with the arid climate of the Late Holocene. Like the 4Akb horizon in KS53, the buried 6Akzb horizons in L11 and L13 represent “reference” soils formed during the HHP. The discrepancy between the sediment age of L16 (6.8 ka) and the older radiocarbon age (TOC) obtained from L15 suggests that older soil material was incorporated into the buried 3Bkzyb (not paleosol) horizon. The inclined and wedge-shaped boundaries between the deeper horizons reflect the dense root network within a nabkah.

##### Laboratory data

The depth functions of the proportions of coarse sand, fine sand, and silt indicate a multilayering, although no clear distinction can be made between runoff and overbank deposits. The silt content in the buried soils (L10-L16) is moderate, ranging from 45 to 65%, while the fine sand content varies between 20 and 40%. The soil is highly salt-affected, with EC values in the paleosol ranging from 4 to 11 dS m^-1^, which is considerably higher than in the overlying cover sediments. Soil pH values in the soils range between 7.9 and 8.3 indicating alkaline conditions. The sulfur content, which is present as calcium sulfate (gypsum), increases from 1.34% at a depth of 45 cm to 3.60% a depth of 60 cm. The organic carbon content of the buried soils is approximately 0.3%. CaCO_3_ contents range between 15 and 30%, with the lowest values found in the gypsum-rich horizons. A relatively high CIA of 70 indicates moderately weathered feldspars.

#### KS5 *Calcic Fluvisol*

The profile KS5 is located south of KS76, near building XI, while KS3 lies about 500 m further north (E 605,166,98, N 2,506,132,76; Figs. 3 and 4). The site currently not affected by lateral erosion but by technical excavation. At present, the area is used for extensive grazing. For pollen analysis, the profile was sampled in 5-cm depth increments, which allowed for higher sampling resolution for radiocarbon dating of the organic soil matter (TOC).

##### 5Akb horizon (L7)

The upper shallow paleosol horizon, which formed in silty and fine-grained runoff deposits (reworked overbank deposit), exhibits a subangular to blocky structure with well-developed aggregates and contains only about 2% coarse material. CaCO_3_ precipitation appears to be weaker than observed in other profiles, but is nevertheless present. Both fine and large filled root channels are visible. The colour of the horizon is dark brown. The upper boundary is sharp, horizontal and straight, the lower boundary is clear. The radiocarbon age (TOC) of the soil organic matter is 5592–5324 cal BP (3639–3501 cal BC).

##### 7Akb horizon (L5)

The lower soil formed within a runoff deposit and consits mainly of silt and fine sand, with coarse material reaching up to 10%. with the proportion of coarse material reaching up to 10%. The soil aggregates occur as subangular blocks, and most aggregates are covered with CaCO₃ precipitation. The soil colour is pale brown. The upper boundary is sharp, horizontal and straight, the lower boundary of this horizon is clear. The radiocarbon age (TOC) of the soil organic matter is 5882–5596 cal BP (3805–3647 cal BC), while the sediment of this layer was dated to 13,255 ± 520 a using OSL.

##### Stratigraphic interpretation

From top to bottom three sediment units can be distinguished (cf. Fig. 5): aeolian deposit (khabra)—runoff deposits—fluvial main-channel deposits. Two paleosol horizons developed on this site: the upper 5Akb horizon, dated to approximately 5600–5300 cal BP, exhibits well-developed aggregates, while the lower 7Akb horizon, which formed around 5900–5700 cal BP shows weaker soil development. Both show no signs of human impact and are therefore natural “reference” soils representing the end of the HHP. The two young dates of organic matter in L1 and L2 (5200–4800 cal BP) can only be explained by the presence of fossil younger roots in a sediment deposited around 13.2/11.5 ka.

##### Laboratory data

The coarse sand fractions are associated with fluvial main-channel deposits and cover sediments. Layers L5 and L7 have a significant sand content (> 20%), while the silt content is 40—50% in the 5Akb horizon and 15–35% in the 7Akb horizon. The buried soils are not salt-affected, with EC values below 0.23 dS m^-1^. The pH values of the paleosols, ranging from 8.2 to 8.9 indicate alkaline conditions. Sulphur is not detectable. The organic carbon content of the buried soils ranges between 0.2 to 0.3%, with higher values (0,44%) recorded in layer L5. CaCO_3_ contents range between 25 and 40% with higher proportions found in the upper soil horizon. CIA values of the selected layers range from 43 to 46, indicating low weathering intensity (< 50).

### Gastropod shells in KS3 as proxy for humid and dry periods in the Early Holocene

Buried soils in fluvial areas often contain materials of varying origins and ages. They frequently harbor relics such as snail shells or plant remains. Although these objects often do not show stratigraphic correlation with the age of the sediments and soils, they can be used to reconstruct the local climate by calculating and observing long-term climate trends.

**Fig. 9:**
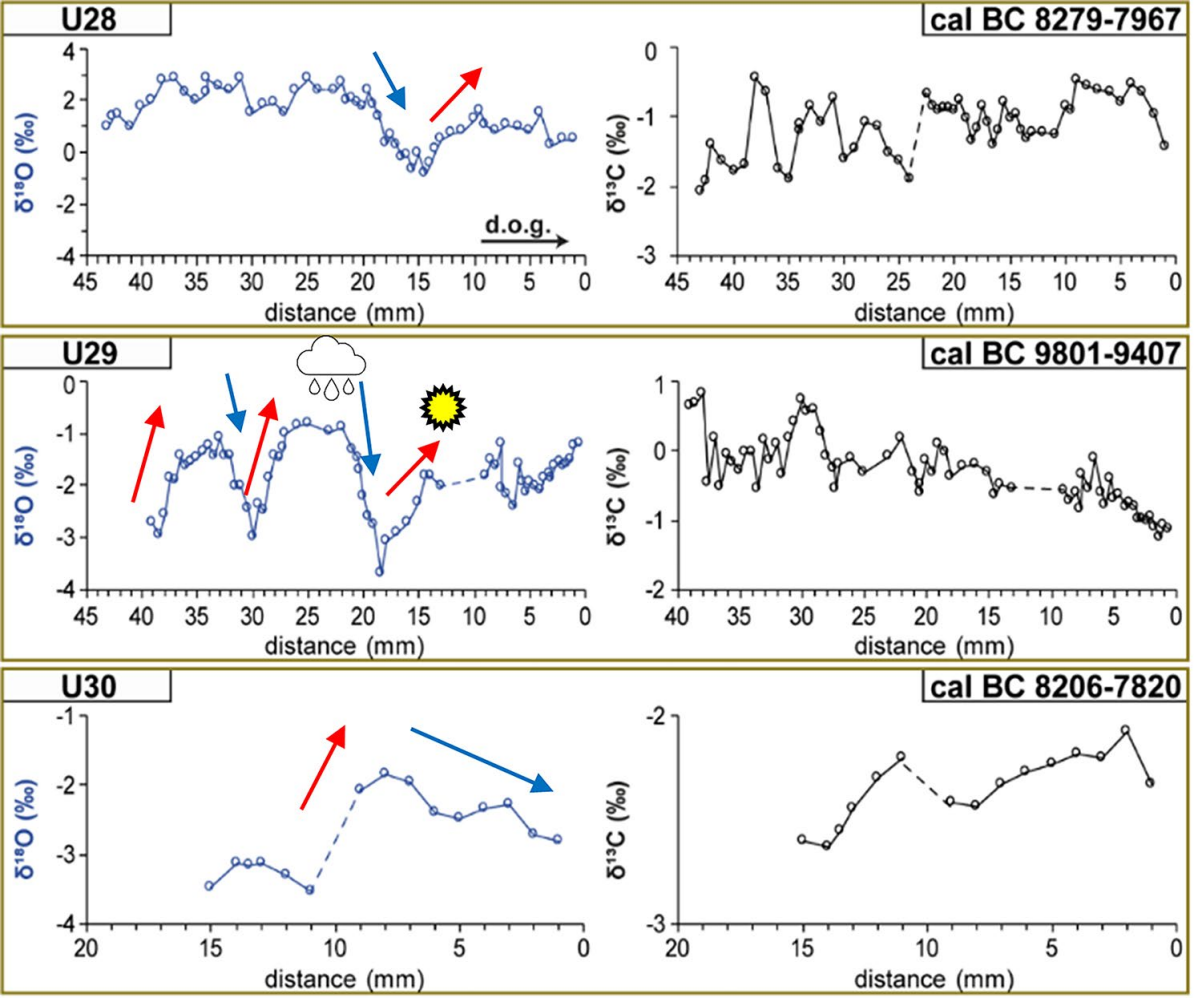
Stable oxygen and carbon isotope data of *Z. insularis* shells from KS3 (U28, U29, U30, Fig. 5; Each circle represents a measured carbon and oxygen isotope data point. Red arrows mark evaporation, blue arrows mark fresh water influx.

In KS3 three *Zootecus insularis* shells (U28, U29, U30) were available for isotopic analyses. Depending on the age and the correlated size of the shell various samples—displayed as circle—were taken per shell (U28 = 56 data points; U29 = 67 data points and U30 = 15 data points, Fig. 9). The aperture is 0 mm, and the direction of growth (d.o.g.) is indicated by an arrow.

A consistent cyclic pattern (alternating between minima and maxima) was observed in all three specimens. However, the width and height of their cycles varied. In shell U28, the cycles were shorter and less pronounced than in the other two shells. Here, the δ^18^O_shell_ values increased from + 1.0 ‰ to + 2.8 ‰. Subsequently, the values fluctuated between + 2.8 ‰ and + 1.5 ‰, forming small cycles, before dropping to -1.0 ‰ (15 mm). Afterward, they recovered slowly until they reached + 2 ‰. Subsequently, they varied between + 2 and + 1 ‰, forming two further cycles. The carbon values exhibited considerable variability, ranging from -2.0 ‰ to + 0.1 ‰. Two distinct cycles were observed in the δ^18^O_shell_ values of shell U29. The first occurred between 38.5 and 30 mm, and the second between 30 and 18.5 mm. The resulting minima were -3.0 ‰ and -3.7 ‰, respectively. The maximum values of the two cycles were -1.1 ‰ and -0.8 ‰, respectively. Following the third minimum, an increase in δ^18^O_shell_ values towards the aperture was observed, with a brief interruption between 8 and 5 mm. The carbon isotope exhibited considerable variability, fluctuating between + 1 ‰ and -1.3 ‰. Following the initial six δ^18^O_shell_ values, shell U30 showed a moderate positive deviation from -3.5 ‰ to—3.1 ‰. Subsequent measurements, taken from 9 mm onwards, showed a significant positive trend, reaching—2.1 ‰ before declining again towards the aperture. The carbon values showed an overall trend to more positive values (from -2.6 ‰ to -2.1 ‰).

Schmitt et al.^45^ interpreted this cyclic pattern as a record of monsoon cycles, illustrating the transition between summer—when evaporation occurs and δ^18^O_shell_ values are more positive—and winter, when freshwater input occurs and δ^18^O_shell_ values are more negative. Since snails exhibit minimal mobility, the observed variation in the δ^18^O shell values can be used as a proxy for reconstructing the local environmental conditions.

However, the role of vegetation in shaping the microenvironment must be taken into account, as increased vegetation density can lead to more shade and consequently to higher humidity. Conversely, it has been shown that a reduction in vegetation leads to a rise in temperature and consequently to increased evaporation. For a detailed discussions please refer to Schmitt et al.^45,52^.

## Synthesis

The investigated buried soils on the left bank of Wadi Shuwayhi at Al-Khashbah were formed primarily from alluvial sediments deposited during two main aggradation phases. Radiocarbon dating, supplemented by cross-dating with optically stimulated luminescence (OSL), indicates that soil formation occurred between 10,600–4400 cal BP, while the youngest soil, the 4Apkzb horizon with an age of 4800–4400 cal BP shows evidence of human-induced alterations. The paleosol horizons, here understood as “undercover proxies” for the HHP, formed up to a maximum of 5300 cal BP. Although the studied profiles represent local phenomena, they may reflect regionally important processes throughout the entire Wadi Samad watershed.

The paleosol horizons share common characteristics: they formed primarily in overbank deposits, while a synergetic soil formation being the most likely process^4^. Most of the soils, whenever being truncated are relics of the past. Pedogenic processes in the HHP were limited to weak aggregation, bioturbation (by roots and soil fauna) and a slight enrichment of soil organic matter with maximum C_org_ values of 0.5%, which is coincident with observations from Yemen^26^.

With the onset of increased aridity and desertification, secondary calcification (CaCO_3_ content > 15–50%) significantly altered the original soil properties. Gypsum precipitation (up to 3.6% S) and enrichment of slightly soluble salts (up to 11 dS/m^-1^, suppl. Table 1) occur in two soils, possibly reflecting pronounced aridification. As a result of this aeolian silty-sandy sediments (khabrah) today cover large parts of the alluvial plains in Southern Arabia^2^ and thus buried soils including paleosol horizons.

### Testing the hypotheses

#### H1

Buried soils that formed under Holocene humid conditions have been preserved within alluvial deposits.

#### H2

Human activities, including cultivation, altered the alluvial deposits at Al-Khashbah during the Early Bronze Age.

#### H3

The stages of soil formation coincide with periods of reduced river activity, characterised by a narrowing of the floodplain, overbank sediment deposition, limited channel shifting, and stabilisation of the wadi banks.

The results confirm H1 by showing that buried soils, represented here primarily by paleosol horizons, formed under humid Holocene conditions and were preserved within alluvial sequences. Soil development differs from that of today (aridification with strong calcification and salinization, while runoff, deflation and desert pavement prevail). Although it remains a challenge to fully correlate soil formation phases with reduced fluvial activity (H3), so far the paleosol horizons clearly coincide with stabilised wadi banks that formed during the two aggradation phases. Similar relationships have been observed in other parts in Oman and the UAE^2,18,19^ or in Tunisia, where buried soils within alluvial sequences represent former stable floodplain surfaces whose alluvial soils developed during phases of reduced flooding and low sedimentation rates^9^.

**Fig. 10:**
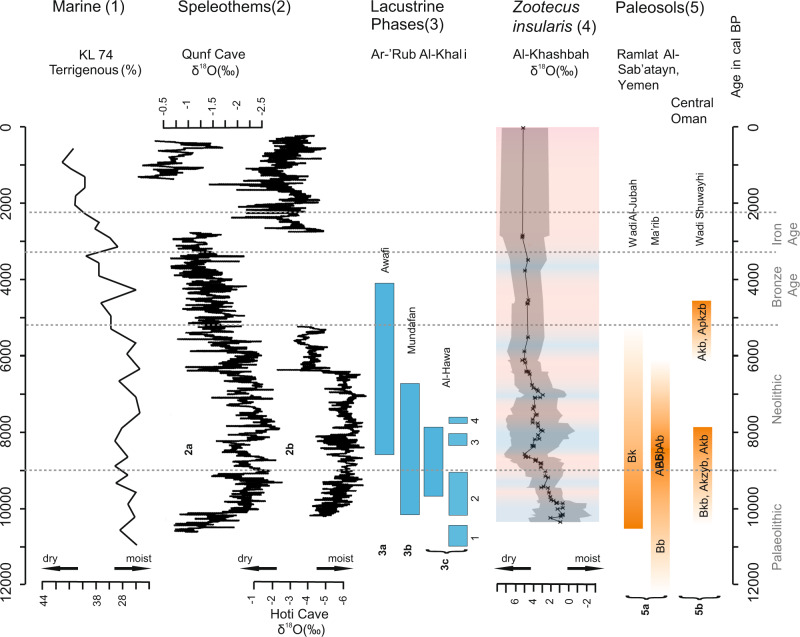
Proxies for Holocene climate change in Southern Arabia, including paleosols and snail shells from Wadi Shuwayhi [Records from the ISM domain in Southern Arabia: (1) KL74 Northern Arabian Sea^31^ (2) (**a**) Qunf Cave, Southern Oman^28^; (**b**) Hoti Cave, Northern Oman^54^; (3) (**a**) Awafi, UAE^55^, (**b**) Mundafan, Saudi Arabia^56^, (**c**) Al Hawa: Ramlat As-Sab’atayn^24^; (4) Central Oman^52^ (5) Wadi Al-Jubah, Bk horizon and not specified paleosol^57^ and Ma’rib^26^. (modified after Pietsch and Kühn 2012^26^).

Our findings from Oman also correspond well with observations from Ma’rib in Yemen (Fig. 10), where buried paleosols formed during geomorphologically stable phases under moderately humid climatic conditions that favoured the establishment of vegetation, leading to the accumulation of organic matter in the soil and the formation of soil aggregates^25–27^. Soil properties, such as low organic carbon and moderate calcium carbonate accumulation, gypsum precipitation, and low weathering indices, indicate limited but distinct soil formation under arid conditions. The same is known from the hyperarid Negev with calcic, salic and gypsic soils on stable alluvial plains from the Holocene and Pleistocene^5,6^.

About 6000 years ago, decreasing rainfall and increasing aridity^24,29,31,32^ coincided with soil improvement and water-harvesting activities by Early Bronze age communities at Al-Khashbah^37^. These activities likely altered soil properties through early horticultural practices. Changes in sediments and soils under irrigation in Iron age oases are well documented^2^, but have not yet been reported from the Bronze Age. Early human settlers tended to settle on stable land surfaces, where they mixed fine sediments through planting activities or soil preparation for agriculture. Wadi terraces and alluvial floodplains thus served as preferred settlement areas^1,14^. The presence of an anthric horizon in KS3 (4Apkzb) at building XI in Al-Khashbah confirmed that cultivation further stabilised the land surface during the Early Bronze Age (H2). The micromorphology revealed a combination of natural pedogenesis and anthropogenic alteration, likely likely associated with early watering practices. Compared to the natural paleosol horizons the soil matrix of the anthric horizon is more homogeneous and the organic matter is not patchily distributed (KS53, KS76).

### Soil formation and environmental context

The studied buried soils formed between 10,600 and 5300 cal BP. The buried alluvial “reference” soil horizons exhibit a subangular blocky macro- and microstructure, bioturbation (strong rooting pattern) and secondary calcification features. The pronounced secondary calcification features indicate alternating dry and moist climatic phases, although the exact sequence remains uncertain. Abrupt stratigraphic boundaries, distinct layering, and truncation of paleosols, as well as the absence of the described pedogenic features in the overlying sediments and in the main-channel fluvial deposits, confirm with a high degree of certainty a significant climatic change during the Holocene in this area. Comparison with the proxies presented clearly shows a correlation between pedogenesis and the Holocene Humid Period (HHP) from 11.5 to 6.5 ka (Fig. 10). In Wadi Shuwayhi paleosols likely formed between 10,600–5300 cal BP, mainly syngenetically with sediment aggradation, as the lower terrace levels correspond to phases of intense overbank deposition^22^.

The arrangement of the fluvial and alluvial landforms in Al-Khashbah according to their elevation (T1, T2 and T3) is generally clear, although several generations of alluvial deposits or cycles of erosion and deposition suggest lateral erosion that has locally cut off older deposits, leaving behind relic buried soils and isolated paleosol horizons. Similar geomorphological complexities have also been documented in other fluvial systems^7,10,14,20^. The relationship between soil formation phases and reduced fluvial activity, as well as river course shifts, remains an important topic for future research, as paleosols in alluvial systems of arid regions serve as valuable proxies of both climatic changes and hydrological processes in the Late Quaternary. Understanding these relationships by including ancient buried soils with signs of human impact deepens our understanding of the Holocene environment and the ways in which ancient human populations adapted to and interacted with arid landscapes^53^.

### Remaining uncertainties

With regard to the completeness of profiles, the exact duration of individual soil formation phases remains unclear, and in areas with high fluvial activity erosion and soil truncation rates continue to pose a major challenge, as also been reported from a comparable watershed in Tunisia^9^. To address this issue, additional profiles across the catchment area and a higher density of chronological data are needed to bridge the temporal gaps between phases of sediment deposition and pedogenesis (KS3 and KS5). In addition, future studies will take a closer look at the Middle Holocene (8 to 6.5 ka), for which only few paleosols have been identified to date.

Another unresolved issue concerns the age discrepancies between snail shells and the surrounding sediment, as well as radiocarbon ages of soil organic matter. Reconstructing the paleoclimate at the local level using terrestrial records is a challenge due to the limited preservation of continuous sediment archives and paleosol sequences. However, the use of the land snail *Zootecus insularis* for geochemical isotope analyses offers a promising approach for investigating both long-term paleoecological changes and short-term hydrological events such as local rainstorms. Although most of the older, intact snail shells that were transported over short distances and subsequently embedded in younger sediments as observed in KS3 (Fig. 5), nevertheless they provide valuable additional information about local climatic fluctuations during the Early Holocene. Unfortunately, these displaced snail shells cannot be used for a detailed stratigraphic interpretation or for reconstructing site-specific sedimentation processes and soil formation.

## Conclusion

The buried soils from Wadi Shuwayhi in Al-Khashbah represent the first paleosol horizons from the HHP in Central Oman. The presence of an anthric horizon KS3 indicates human activity dating back to the Early Bronze Age around 4800–4400 cal BP. Sediment and soil records from the left bank of Wadi Shuwayhi at Al-Khashbah provide new insights into paleosol development within a fluvial/alluvial system in an arid region. The buried soils reflect periods of surface stability associated with moister climate conditions than today’s, and they demonstrate the interplay between overbank deposition (aggradation), early agricultural activities, and landscape development. Soil formation during the HHP predominantly occurred during geomorphologically stable phases, visible in the aggradation of silty sediments, wadi bank stabilisation and the establishment of vegetation.

Soil properties such as low soil organic carbon content, the precipitation of calcium carbonate and gypsum, and low values of weathering indices together indicate limited but verifiable soil formation under semi-arid conditions. Stable isotope data from terrestrial gastropods further suggest fluctuating hydrological conditions during the Early Holocene, characterised by alternating moist and dry phases associated with variations in the monsoon. These biological proxies underscore the sensitivity of the landscape to climatic oscillations.

Taken together, the combined stratigraphic, micromorphological, geochemical and paleontological data from Al-Khashbah demonstrate that the HHP facilitate both phases of soil formation within a dynamic fluvial environment. The subsequent aridification is reflected in the transition from alluvial to aeolian sedimentation, as well as the occurrence of surface sealing. Buried paleosol horizons in the terrace fillings, serve as important archives for reconstructing past local environmental conditions and human–environment interactions in arid alluvial plains.

## Supplementary Information

Below is the link to the electronic supplementary material.

Supplementary Material 1

## Data Availability

The supplementary file can be found as suppl. Table 1 and 2 and more data from the BMBF project are available at 10.5334/oq.153.s1.

## Declarations

### Sampling permission

All the work including the export of samples was carried out with the approval of the Ministry of Heritage and Tourism in the Sultanate of Oman. We are very grateful for their support.
