# Correction: Buried soils from the Holocene Humid Period in Wadi Shuwayhi, Al-Khashbah (Oman)

**DOI:** 10.1038/s41598-026-63975-1

**Published:** 2026-07-28

**Authors:** Dana Pietsch, Tara Beuzen-Waller, Conrad Schmidt, Katharina E. Schmitt, Peter Kühn

**Affiliations:** 1 Research Area Geography, Chair of Soil Science and Geomorphology, University of Tübingen, Tübingen, Germany; 2 Institute of Geography, University of Heidelberg, Heidelberg, Germany; 3 UMR 7194 HNHP—PAST, University of Perpignan Via Domitia, Perpignan, France; 4 Institute for Ancient Near Eastern Studies (IANES), University of Tübingen, Tübingen, Germany; 5 Institute of Geosciences, Applied and Analytical Paleontology Work Group, Johannes Gutenberg University Mainz, Mainz, Germany

Correction to: *Scientific Reports* 10.1038/s41598-026-55740-1, published online 07 July 2026

The original version of this Article contained errors in Figures 7 and 8, and Reference list.

Firstly, Figure 7 was a duplicated version of Figure 8 and both Figures’ Legends were incorrect.

As a result, the Legend of Figure 7,

“Fig. 7. Micromorphological features of KS3 anthric horizon 4Apkzb.”

now reads:

“Fig. 7. Micromorphological features of KS3 anthric horizon 4Apkzb. (L8) upper photograph – plane polarised light: subangular blocky microstructure and voids (v); coarse material consists of fresh limestone fragments, alterites and rounded bone fragments; dark grey areas represent micritic calcium carbonate masses; lower photograph – plane polarised light: crumb micro-structure; speckled light grey areas are pedogenic gypsum precipitations.”

In Addition, the Legend of Figure 8,

“Fig. 8. Micromorphological features of KS3 paleosol horizon 5Bkb (L8) upper photograph – plane polarised light: subangular blocky mi- (L7) upper photograph—plane polarized light: Subangular blocky to crostructure; coarse material consists of fresh limestone fragments, al- crumb microstructure, dark grey areas represent micritic carbonate terites and rounded bone fragments; dark grey areas represent micritic precipitations and hypocoatings (mc) around voids; Lower photograph—calcium carbonate masses; lower photograph—plane polarised light: crossed polarised light: subangular blocky to crumb microstructure with crumb microstructure; speckled light grey areas are pedogenic gyp- crystallitic b-fabric in peds; root residues in voids (v) with associated sum precipitations tabular to lenticular gypsum precipitations (g).”

now reads:

“Fig. 8. Micromorphological features of KS3 paleosol horizon 5Bkb (L7) upper photograph — plane polarised light: subangular blocky crumb microstructure, dark grey areas represent micritic carbonate precipitations and hypocoatings (mc) around voids; lower photograph — crossed polarised light: subangular blocky to crumb microstructure with crystallitic b-fabric in peds; root residues in voids (v) with associated tabular to lenticular gypsum precipitations (g).”

The correct Figure 7 is now updated in the original article.

In Addition, Reference 38,

“38. De Gramont, X. Geological map, Oman 1:100,000 . sheet NF 40–7C . Șamad / Sultanate of Oman, Ministry of Petroleum and Minerals, Directorate General of Minerals ; geological mapping By X. de Gramont ... [et al.] ; computer-assisted cartography by cartography department Bureau de recherches geologiques et minires, Orleans, France - Sorbonne Universite. https://primo.sorbonne-universite.fr/discovery/fulldisplay/alma991001358029806616/33BSU_INST:33BSU.”

now reads:

“38. De Gramont, X. Geological map, Oman 1:100,000 . sheet NF 40–7C . Sultanate of Oman, Ministry of Petroleum and Minerals, Directorate General of Minerals ; Geological mapping By X. de Gramont ... [et al.]; Computer-assisted cartography by cartography department Bureau de recherches geologiques et minires, Orleans, France - Sorbonne Universite. https://primo.sorbonne-universite.fr/discovery/fulldisplay/alma991001358029806616/33BSU_INST:33BSU.”

Finally, Reference 42,

“42. Govindaraju, K. Special issue of geostandards newsletter. Geostandards Newsletter 44 (1994).”

now reads:

“42. Govindaraju, K. Special issue of geostandards newsletter. Geostandards Newsletter **44** (1994).”

The original Article has been corrected.

