# Supplementary material for: Buried soils from the Holocene Humid Period in Wadi Shuwayhi, Al-Khashbah (Oman)
Source: Sci Rep. 2026 Jul 7;16:21215. doi: 10.1038/s41598-026-55740-1 (PMC13346906; doi:10.1038/s41598-026-55740-1)
Supplement: Supplementary file 1 — Supplementary Material 1 [file 41598_2026_55740_MOESM1_ESM.pdf]

Profile-layer	Sample number	Soil horizon	Depth [cm]	EC [µS/cm]	pH $_{H2O}$	$CaCO_3$	C_t	S_t	C_{org}	0,01 - 2 µm	2 - 50 µm	50 - 200 µm	200 - 500 µm	500 - 2000 µm	CIA*
						[%]									
KS3-11	U459-1	C	0-5	105	9.1	11.2	1.34	< 0.05	0.00	1.0	7.9	7.8	22.1	61.3	
KS3-10	U458-1	2C	5-8	244	8.7	21.6	2.59	< 0.05	0.00	9.2	50.6	14.7	4.7	20.8	
KS3-9	U457-1	3C	8-15	1320	8.2	26.7	3.32	0.14	0.12	7.2	49.3	21.8	4.8	16.9	41.6
KS3-8	U456-1	4Apkzb	15-20	8280	8.0	25.8	3.28	0.36	0.19	8.9	57.3	11.3	7.2	15.3	46.4
KS3-8	U455-1	4Apkzb	20-30	4610	8.1	25.3	3.30	0.62	0.27	9.8	72.9	13.0	3.3	0.9	47.6
KS3-8	U454-1	4Apkzb	30-36	4890	8.2	29.2	3.68	0.60	0.18	10.5	75.7	9.9	2.9	1.0	48.5
KS3-7	U453-1	5Bk	36-45	2780	8.5	38.4	4.77	0.13	0.17	10.1	61.4	6.9	1.2	20.4	48.3
KS3-7	U452-1	5Bk	45-50	2400	8.5	33.1	4.20	0.13	0.23	10.8	73.8	6.3	2.4	6.7	49.6
KS3-6	U451-1	6C	50-58	1653	9.0	23.3	2.92	0.08	0.12	10.4	65.1	10.3	7.5	6.6	
KS3-5	U450-1	7C	58-60	891	9.5	14.0	1.70	< 0.05	0.02	6.3	53.3	23.5	7.2	9.7	
KS3-4	U467-1	8C	60-65	877	9.6	14.1	1.60	< 0.05	0.00	2.3	24.2	16.6	10.1	46.9	
KS3-4	U466-1	8C	65-70	760	9.7	13.0	1.54	< 0.05	0.00	7.1	62.2	19.1	9.6	2.0	
KS3-3	U465-1	9C	70-80		9.9	9.3	1.05	< 0.05	0.00	2.8	22.7	21.1	9.2	44.2	
KS3-3	U464-1	9C	80-90	347	10.0	7.7	0.86	< 0.05	0.00	1.0	8.9	9.1	20.5	60.5	
KS3-2	U463-1	10C	90-100	318	9.9	9.4	1.04	< 0.05	0.00	0.1	3.2	3.9	23.6	69.2	
KS3-1	U462-1	11C	100-110	239	9.9	8.8	1.00	< 0.05	0.00	0.2	2.0	1.4	13.6	82.8	
KS3-1	U461-1	11C	110-120	174	9.8	8.2	0.78	< 0.05	0.00	1.8	12.0	6.2	15.7	64.2	
KS53-17	U1204-1	C	30-40	516	7.9	32.7	4.10	0.14	0.17	12.5	70.2	11.6	2.5	3.1	
KS53-17	U1205-1	C	40-50	601	8.0	41.1	5.14	0.06	0.20	10.7	61.3	25.7	1.8	0.5	
KS53-16	U1206-1	2C	50-60	933	8.2	45.5	5.65	0.06	0.19	10.2	61.1	26.6	1.9	0.1	
KS53-16	U1207-1	2C	60-70	1454	8.5	45.0	5.89	0.07	0.49	10.2	68.1	21.2	0.6	0.0	
KS53-16	U1208-1	2C	70-80	1655	8.7	45.8	5.68	0.06	0.18	12.0	64.9	23.0	0.0	0.0	
KS53-15	U1209-1	3C	80-90	2680	8.6	49.4	6.20	0.10	0.27	12.9	59.2	9.5	6.0	12.3	72.2
KS53-14	U1210-1	4Akb	90-100	2610	8.7	32.0	3.99	0.09	0.16	8.7	64.1	20.1	4.3	2.9	74.6
KS53-13	U1211-1	5Bkmb	100-110	2010	8.7	32.6	3.87	0.08	0.00	5.0	47.3	34.0	8.2	5.5	75.5
KS53-13	U1212-1	5Bkmb	110-120	1906	8.7	31.5	3.77	0.05	0.00	7.7	67.4	22.8	2.0	0.1	75.4
KS53-13	U1213-1	5Bkmb	120-130	1805	8.6	32.4	3.98	< 0.05	0.09	7.3	69.6	20.8	2.2	0.2	74.7
KS53-13	U1214-1	5Bkmb	130-140	1921	8.5	30.9	3.69	< 0.05	0.00	8.4	68.8	8.3	6.6	8.0	75.6
KS53-12	U1215-1	6C	140-150	715	8.9	10.9	1.19	< 0.05	0.00	1.9	13.9	9.0	11.4	63.8	
KS53-12	U1216-1	6C	150-160	433	9.1	9.5	0.98	< 0.05	0.00	2.7	17.0	15.3	33.5	31.6	
KS53-12	U1217-1	6C	160-170	213	8.9	9.9	1.01	< 0.05	0.00	2.1	11.3	10.6	10.6	65.4	
KS53-11	U1218-1	7C	170-180	198	8.7	25.3	3.01	< 0.05	0.00	9.6	57.3	7.6	3.2	22.4	
KS76-18	U2013-1	C	0-5	249	7.5	13.01	2.22	< 0.05	0.66	0.9	9.8	28.0	40.2	21,0	
KS76-18	U2014-1	C	5-10	295	7.6	17.53	2.44	< 0.05	0.33	1.3	13.8	39.0	35.7	10,2	
KS76-18	U2015-1	C	10-15	168	7.9	15.82	2.35	< 0.05	0.45	1.1	10.6	25.6	18.5	44,3	
KS76-18	U2016-1	C	15-20	180	8.1	15.49	2.11	< 0.05	0.25	2.4	14.1	22.7	20.4	40,5	
KS76-17	U2017-1	2C	20-25	1063	7.9	15.82	2.17	< 0.05	0.27	10.2	61.1	26.7	2.0	0,0	
KS76-16	U2018-1	3Bkzyb	25-30	2690	8.0	19.52	2.53	0.06	0.19	8.8	65.8	22.4	2.5	0,5	
KS76-16	U2019-1	3Bkzyb	30-35	3990	8.0	20.63	2.78	0.07	0.30	6.5	55.0	27.0	6.9	4,6	
KS76-16	U2020-1	3Bkzyb	35-40	5460	8.0	21.21	2.83	0.09	0.29	7.2	50.9	26.2	8.6	7,0	
KS76-16	U2021-1	3Bkzyb	40-45	7880	7.9	19.38	2.67	0.33	0.35	6.5	64.8	24.2	3.9	0,7	
KS76-16	U2022-1	3Bkzyb	45-50	9280	8.0	15.98	2.21	1.34	0.29	4.3	45.0	22.9	13.0	14,8	
KS76-16	U2023-1	3Bkzyb	50-55	9800	8.0	15.10	2.04	2.22	0.23	5.8	54.5	21.4	3.6	14,8	
KS76-16	U2024-1	3Bkzyb	55-60	9950	8.1	13.98	1.91	3.60	0.23	7.4	69.2	22.0	1.4	0,2	
KS76-15	U2025-1	4Akzyb	60-65	10180	8.1	16.27	2.24	1.25	0.28	4.2	50.4	35.6	8.7	1,0	

KS76-15	U2026-1	4Akzyb	65-70	11140	8.1	17.72	2.30	1.67	0.17	4.3	47.8	34.6	7.1	6,2	
KS76-14	U2027-1	5Bkzyb	70-75	11150	8.1	18.31	2.33	1.68	0.14	5.6	57.8	30.6	5.0	1,0	68.8
KS76-14	U2028-1	5Bkzyb	75-80	10490	8.1	20.31	2.68	0.69	0.24	4.3	45.7	40.4	7.6	2,0	62.9
KS76-14	U2029-1	5Bkzyb	80-85	9920	8.2	24.10	3.19	0.17	0.29	5.4	51.7	36.9	5.5	0,5	61.8
KS76-14	U2030-1	5Bkzyb	85-90	8900	8.3	26.79	3.51	0.09	0.30	5.6	49.6	35.4	8.3	1,2	67.2
KS76-13	U2031-1	6Akzb	90-95	8750	8.3	24.49	3.35	0.09	0.41	6.8	62.6	30.0	0.7	0,0	67.0
KS76-13	U2032-1	6Akzb	95-100	8520	8.0	26.34	3.38	0.07	0.22	5.2	49.9	31.7	2.3	10,9	67.9
KS76-13	U2033-1	6Akzb	100-105	9410	8.1	29.54	3.87	0.07	0.32	6.6	52.8	35.3	4.9	0,3	66.9
KS76-12	U2034-1	6Ckm	105-110	6910	8.3	28.65	3.60	0.05	0.16	5.0	48.9	36.7	8.5	0,9	69.3
KS76-11	U2035-1	6Akzb2	110-115	8930	8.2	24.52	3.08	< 0.05	0.13	4.1	43.9	33.1	13.6	5,3	68.5
KS76-10	U2036-1	7Bkzb	115-120	9040	8.2	23.59	2.93	0.05	0.10	5.3	51.4	29.0	7.1	7,2	68.1
KS76-10	U2037-1	7Bkzb	120-125	8600	8.2	25.25	3.15	0.05	0.12	5.1	44.0	22.5	9.3	19,1	68.1
KS76-10	U2038-1	7Bkzb	125-130	7670	8.3	25.58	3.27	0.07	0.20	3.4	28.4	11.6	6.8	49,7	65.7
KS76-10	U2039-1	7Bkzb	130-135	9430	8.2	26.98	3.45	0.07	0.21	3.9	36.5	16.9	6.7	36,0	67.0
KS76-9	U2040-1	8C	135-140	8050	8.3	27.38	3.42	0.06	0.14	4.5	50.7	34.4	7.2	3,3	68.7
KS76-9	U2041-1	8C	140-145	6480	8.3	23.06	2.98	0.05	0.22	4.7	49.9	33.9	6.1	5,4	71.0
KS76-9	U2042-1	8C	145-150	6440	8.3	16.81	2.34	< 0.05	0.33	4.5	52.2	31.0	7.3	5,0	61.8
KS5-10	U67-1	9C	0-5	80	8.9	12.1	1.57	< 0.05	0.12	1.7	12.0	26.8	27.9	31.6	
KS5-10	U66-1	9C	5-10	69	8.7	14.2	1.59	< 0.05	0.00	1.2	8.2	15.1	17.1	58.4	
KS5-9	U65-1	C	10-15	92	9.1	12.5	1.52	< 0.05	0.02	7.4	21.9	31.6	27.8	11.3	
KS5-9	U64-1	C	15-20	99	8.8	13.0	1.50	< 0.05	0.00	6.5	23.9	23.8	13.4	32.4	46.0
KS5-8	U63-1	2C	20-25	102	9.0	13.2	1.54	< 0.05	0.00	4.3	23.8	36.1	27.3	8.5	45.2
KS5-8	U62-1	2C	25-30	148	8.5	16.9	2.12	< 0.05	0.09	7.5	33.3	8.8	11.2	39.1	
KS5-8	U61-1	2C	30-35	151	8.9	19.9	2.61	< 0.05	0.22	15.4	65.7	11.1	3.7	4.2	
KS5-8	U60-1	2C	35-40	153	8.5	20.4	2.68	< 0.05	0.23	11.8	65.9	9.5	9.7	3.1	45.2
KS5-7	U59-1	5Akb	40-45	169	8.9	21.3	2.76	< 0.05	0.20	6.9	42.6	22.3	0.0	28.3	43.4
KS5-7	U58-1	5Akb	45-50	138	8.5	20.8	2.55	< 0.05	0.06	7.5	48.8	19.3	0.1	24.3	44.4
KS5-6	U57-1	6C	50-55	136	9.0	21.7	2.53	< 0.05	0.00	9.9	59.4	30.7	0.0	0.0	
KS5-6	U56-1	6C	55-60	129	8.9	18.8	2.57	< 0.05	0.32	7.4	52.9	39.7	0.0	0.0	
KS5-5	U55-1	7Akb	60-65	178	8.3	16.9	2.32	< 0.05	0.30	4.7	34.3	25.5	12.0	23.5	44.1
KS5-5	U54-1	7Akb	65-70	235	8.6	16.3	2.39	< 0.05	0.44	2.1	14.9	6.9	2.1	74.0	45.0
KS5-4	U53-1	8C	70-75	191	9.0	17.6	2.35	< 0.05	0.24	1.4	9.8	5.1	0.5	83.2	
KS5-4	U52-1	8C	75-80	177	8.7	16.2	2.17	< 0.05	0.23	2.6	18.2	6.1	5.2	67.9	
KS5-3	U51-1	9C	80-85	236	9.0	16.2	2.07	< 0.05	0.12	5.5	44.1	16.5	8.6	25.3	
KS5-3	U50-1	9C	85-90	110	8.8	13.6	1.84	< 0.05	0.20	0.9	8.3	3.9	0.7	86.2	45.0
KS5-3	U49-1	9C	90-95	101	9.2	12.9	1.72	< 0.05	0.17	5.0	43.1	30.3	15.8	5.7	
KS5-3	U48-1	9C	95-100	152	8.8	15.0	1.91	< 0.05	0.11	6.3	45.6	13.3	4.1	30.8	

Table 1: Laboratory data of sediments and paleosols (A horizons are highlighted in dark grey, B Horizons in light grey; *Chemical index of alteration

Lab number	Sample number	Site-layer	OSL age [a]	±	a BP (uncalibrated)	a cal BP (2σ)	a cal BC/AD (2σ)
Poz-169324	U227-3	KS3-11			2050±30	2108-1925	159–26 cal BC-AD
MAMS 61245	U30	KS3-9 snail			8852±28	10155-9769	8206-7820 cal BC
MAMS 61244	U29	KS3-8 snail			10053±30	11750-11356	9801-9407 cal BC
MAMS 61243	U28	KS3-8 snail			8962±30	10227-9916	8279-7967 cal BC
Poz-174588	U2094-1	KS3-8			4105±35	4817-4450	2868–2501 cal BC
BG 5612	U2092-1	KS3-8	8065	225			
Poz-174641	U2093-1	KS3-7			7510±170	8689-7961	6740–6012 cal BC
BG 5632	U2288-1	KS53-15	9585	690			
Poz-174638	U1146-1	KS53-14			9080±160	10657-9697	8708–7748 cal BC
BG 5631	U2287-1	KS53-12	10320	1085			
BG 5630	U2286-1	KS53-11	22635	2360			
Poz-168944	U2827-1	KS76-18			1705±30	1698-1533	252–418 cal AD
Poz-169327	U2828-1	KS76-17			3990±35	4570-4303	2621–2354 cal BC
Poz-169329	U2833-1	KS76-16			6810±50	7742-7573	5793–5624 cal BC
BG 5620	U2167-1	KS76-16	6915	345			
Poz-169328	U2835-1	KS76-15			7380±50	8330-8035	6381–6086 cal BC
Poz-174640	U2011-1	KS76-13			7420±60	8372-8039	6423–6090 cal BC
Poz-174637	U2010-1	KS76-11			7560±60	8512-8194	6563–6245 cal BC
Poz-169313	U2838-1	KS76-10			7270±60	8189-7965	6240–6016 cal BC
Poz-169314	U2841-1	KS76-9			7420±20	8372-8039	6423–6090 cal BC
BG 5619	U2166-1	KS76-1	11495	1045			
Poz-178140	U2100-1	KS5-9			3540±35	3957-3699	1971–1750 cal BC
Poz-178139	U2099-1	KS5-7			4760±40	5592-5324	3639–3501 cal BC
Poz-178137	U2098-1	KS5-5			4970±40	5882-5596	3805–3647 cal BC
BG 5613	U2096-1	KS5-5	11460	680			
BG 5614	U2097-1	KS5-3	13255	520			
Poz-169326	U2821-3	KS5-2			6080±50	7152-6792	5208–4843 cal BC
Poz-168943	U2823-3	KS5-1			6150±80	7254-6799	5305–4850 cal BC
Poz-169010	U2825-3	KS5-1			9400±80	11070-10309	9121-8360 cal BC

Table 2: Radiocarbon and OSL dating results (Calibration of radiocarbon data with Oxcal 4.4 [47] using IntCal20 [48]; Luminescence Dose and Age Calculation (LDAC) platform for OSL [51]
